# Investigation of Cellulose-Based Materials Applied in Life Sciences Using Laser Light Scattering Methods

**DOI:** 10.3390/polym16081170

**Published:** 2024-04-21

**Authors:** Anca-Giorgiana Grigoras

**Affiliations:** “Petru Poni” Institute of Macromolecular Chemistry, Grigore Ghica Voda Alley, 41A, 700487 Iasi, Romania; angrig@icmpp.ro

**Keywords:** laser light scattering, cellulose-based materials, particle size distributions, molecular weight distributions, conformation

## Abstract

This review emphasizes the practical importance of laser light scattering methods for characterizing cellulose and its derivatives. The physicochemical parameters like molecular weights, the radius of gyration, hydrodynamic radius, and conformation will be considered when the reproducibility of polymer behavior in solution is necessary for the subsequent optimization of the property profile of a designed product. Since there are various sources of cellulose, and the methods of cellulose extraction and chemical modification have variable yields, materials with variable molecular weights, and size polydispersity will often result. Later, the molecular masses will influence other physicochemical properties of cellulosic materials, both in solution and solid state. Consequently, the most rigorous determination of these quantities is imperative. In this regard, the following are presented and discussed in this review: the theoretical foundations of the light scattering phenomenon, the evolution of the specific instrumentation and detectors, the development of the detector-coupling techniques which include a light scattering detector, and finally, the importance of the specific parameters of polymers in solution, resulting from the data analysis of light scattering signals. All these aspects are summarized according to the chemical classification of the materials: celluloses, esters of cellulose, co-esters of cellulose, alkyl esters of cellulose, ethers of cellulose, and other heterogeneous cellulose derivatives with applications in life sciences.

## 1. Introduction 

Cellulose is a biopolymer obtained from wood (*Pinus* sp. and *Eucalyptus* sp.), plants (cotton, sisal, and ramie), agricultural byproducts (soy hulls, rice husk, and corncob), tunicate (*Ascidiacea* sp. and *Halocynthia* sp.), algae (*Micrasterias* sp., *Valonia* sp., *Caldophora* sp., and *Boergesenia* sp.), or bacteria (*Acetobacter* sp.) [1,2,3,4,5]. In terms of structure, it comprises ordered crystalline regions and amorphous regions in a variable ratio depending on the cellulose source. Even though these domains are governed by van der Waals interactions and hydrogen bonds, and determine the formation of a cohesive network, the amorphous regions are considered structural defects easily accessible to a limited range of acidic solvents.

Because the intramolecular and intermolecular hydrogen bonds represent an impediment to the dissolution of pure cellulose in either cold or hot water, approximately 35% of the purified cellulose is chemically converted into cellulose derivatives, mainly as esters and ethers of cellulose.

Humans consume a wide range of biomaterials in daily life. Pharmaceuticals, cosmetics, and food products contain cellulose and cellulose derivatives as biocompatible materials, which are physiologically harmless and well-tolerated by the mucous membranes and skin [6,7]. Also, these polysaccharides are integrated into many industries and fulfill the role of suspending agents, antimicrobial agents, thickeners, emulsion stabilizers, defoaming surfactants, additives, binders in coatings, or absorbents of pollutants. The broadening of the biomedical applications allowed the design of antimicrobial hydrogels, hydrogels for tissue engineering, and hydrogels for food additives or food packaging based on cellulose [8,9,10,11]. The production of pharmaceutical textiles based on cellulose is supposed to functionalize the polysaccharides with plant-derived materials, drugs, and metal nanoparticles [12]. Additionally, cellulose-based materials as superabsorbent hydrogels contribute to pollution control and water treatment [13,14,15], or are used as supercapacitors, batteries, and chemical/biological/physical sensors in the energy and sensing industries [16,17].

The solution properties of cellulose-based materials are influenced by the chemical structure and substitution degree as well as by the molecular masses, the radius of gyration, the hydrodynamic radius, and their distributions. These parameters are taken into account when the reproducibility of polymer behavior in solution is necessary for the optimization of the property profile of a designed product. Kulicke et al. tested different coupled techniques for separation in the solution of water-soluble cellulose derivatives. Besides average molar mass, they obtained molar mass and size distributions [18].

Since cellulose-based materials cannot melt, and their dissolution became a major issue, researchers were constrained to test different solvent systems. If the polymer coils are well dispersed in solution at the molecular level and no form aggregates, the distribution functions of the size and molecular masses of derivatives could be determined by detector-coupling techniques involving a light scattering detector [19].

Several review articles have discussed various aspects of the structure, properties, and applications of materials based on cellulose. The crystallinity of cellulosic materials was studied using multiple methods: X-ray diffraction, nuclear magnetic resonance, Fourier transform-infrared spectroscopy, and Raman spectroscopy. In bulk, it was observed that the crystallinity degree and crystallite size of cellulose influence the thermal stability and morphology of materials. Even if cellulosic materials are used in the final applications as powders, films, or gels, the characterization of their solutions is equally important. Often, in solution, the nature of the solvent is the main considered factor [1,20,21,22,23].

This work covers the specific topic of characterization in solution of cellulose-based materials and emphasizes the practical importance of the laser light scattering methods.

## 2. Theory of the Light Scattering Phenomena

Static light scattering (SLS) and dynamic light scattering (DLS) represent methods of studying polymers based on the fact that the light from a laser source that passes through a homogeneous medium (e.g., a polymer solution) will be scattered in all directions. The intensity of scattered light is recorded at different angles *θ* to the direction of the beam incident on the sample. It is correlated with other physical quantities characteristic of macromolecular chains in a solution, like the weight-average molecular weight *M_w_*, the second virial coefficient *A*_2_, the radius of gyration *R_g_*, and the hydrodynamic radius *R_h_*. Then, based on information about the nature and size of macromolecules, and the strength of interactions present in solution, various types of molecular complexes and phenomena of aggregation, association, or micellization can be analyzed.

### 2.1. Static Light Scattering

Static/elastic/Rayleigh/classical scattering of light occurs at a wavelength value equivalent to the value of incident light. It refers to an experiment in which light intensity is determined at a scattering angle by averaging fluctuated intensities on a large time scale compared with a temporal scale of the intensity fluctuations. The basic equation that describes the SLS phenomenon, and connects the scattering light intensity with the properties of macromolecules larger than *λ*_0_/20 present in solution, is called the Zimm equation:

*K* × *c/R_θ_* = 1/*M_w_* × *P*(*θ*) + 2*A*_2_ × *c/P*(*θ*) + 3*A*_3_ × *c*^2^/*P*(*θ*) (1)

with

*K* = (4*π*^2^ *n*^2^/*λ*_0_^4^ *N_A_*)(*dn/dc*)^2^ (2)

and

*P*(*θ*) = 1 − (16*π*^2^ *R_g_*^2^ sin^2^(*θ/*2)/3*λ*_0_^2^) (3)

where *K* is the optical constant for vertically polarized incident light, *c* is the concentration of scattering species (in g/mL), *R_θ_* is the Rayleigh ratio between intensities of incident and scattered light at *θ* angle, also named the excess Rayleigh ratio (in cm^−1^), *M_w_* is the weight-average molecular mass of particles in solution, *n* is the refractive index of the solution, *N_A_* is the Avogadro number, *λ*_0_ is the wavelength of light incident on the sample, and *A*_2_ and *A*_3_ are the virial coefficients [24]. The incident light for scattering measurements may come from a GaAs laser, HeNe laser, or Ar^3+^ laser which operates at *λ*_0_ = 658 nm, 632.8 nm, or 514.5 nm, respectively. The theoretically derived form factor *P*(*θ*) is a function of the *z*-average size, shape, and structure of the molecule.

In the case of a polymer, the radius of gyration *R_g_* can be defined in terms of a distribution of distances *r_i_* (in either direction) for each monomer, measured from the gravity center of the macromolecule:

(4) $$R_{g}^{2}=\sum_{i}r_{i}^{2}$$

The molecules with different conformations (compact spheres, rods, disordered coils, etc.) have different radii of gyration *R_g_*. To obtain an experimental *R_g_* means the opportunity to determine molecular structure. Between *R_g_* and dimensions characteristic of different macromolecular patterns the following relationships exist:

*R_g_*^2^ = 3 *R*^2^/5 (compact sphere with radius *R*) (5)

*R_g_*^2^ = *L*^2^/12 (rod with length *L*) (6)

*R_g_*^2^ = *α*^2^ *a*^2^ *M*/6 *M*_0_ (flexible/random coil) (7)

*R_g_*^2^ = *R*^2^ (hallow sphere/spherical shell with radius *R*) (8)

where *α* is the expansion factor, *M*_0_ is the molecular weight of the Kuhn segment, and *M* is the molecular weight of the polymer. The macromolecule shape in a solution can be appreciated by calculating *R_g_* values based on previous relationships and comparing them with the experimental values from light scattering measurements. As there is a variety of structural models, the experimental determination of *R_g_* does not allow the immediate award of a specific conformation. However, the light scattering measurements lead to a restriction of types of proposed structural models. Some accurate conclusions about the actual conformation of the macromolecule in a given system are drawn only through comparison with experimental results from other methods (viscometry or X-ray diffraction).

In practice, there are nanoparticles with ellipsoidal shapes in suspension, too. The monodisperse spheroids have a specific diameter of *x* and a length of *L*. The spheroids with polydispersity in both length and diameter can be mathematically treated with the Rayleigh–Gans–Debye theory of light scattering. The new light scattering methodology is a quick and robust tool for the size characterization of polydisperse spheroidal nanoparticles. The polydispersity indices, obtained by fitting the theoretical form factor *P(θ)* to experimental data, allow the determination of average dimensions (weight-average diameter *x_w_* and weight-average length *L_w_*):

(*L_w_*)^3^ − (20<*r*^2^>*_z_ L_w_*)/*X*_2_ + 12*M_w_* (A + 1)(*X*_1_/*X*_2_)/*πηρN_A_* (A + 2) = 0 (9)

(*x_w_*)^2^ = 6 *M_w_* (A + 1)/*πηρN_A_L_w_* (A + 2) (10)

In Equations (9) and (10), the term *ρ* stands for the density of the particles with the molecular weight *M*, *η* represents the packing density of the spheroids in aggregates, *X*_1_ = (A + 3)(A + 2)/(A + 1)^2^, *X*_2_ = (C + 3)(C + 2)(C + 1)^2^, A = 1/(*P*_x_ − 1), and C = 1/(*P_L_* − 1), where A and C are the polydispersity parameters for the diameter and length [25]. The parameters <*r*^2^>*_z_* and *M_w_* can be directly extracted from the light scattering experiments.

Generally, the mean square radius <*r*^2^> refers to a single particle, while <*r*^2^>_z_ is the *z*-average mean square radius for the random coils of particles in a theta solvent. If we assume that the particle size distribution is essentially monodisperse, and the particles have the same mean square radius <*r*^2^>, then the root mean square RMS radius (also named the radius of gyration *R_g_* in practice) is defined as the square root of the mean square radius according to the following equation:

*R_g_* = <*r*^2^>^1/2^ or *R_g_*^2^ = <*r*^2^> (11)

**Figure 1:**
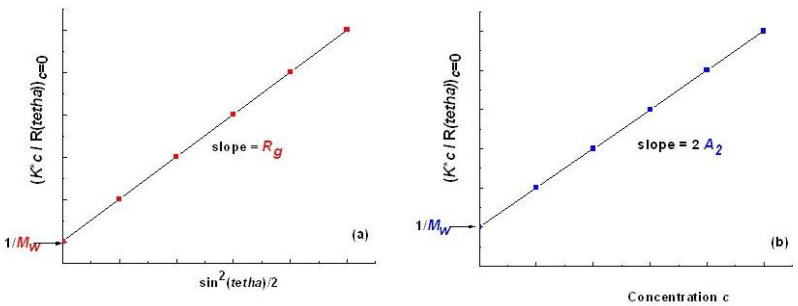
Graphical representation of Zimm equation for limit cases: (**a**) *c* = 0 and (**b**) *θ* = 0 (adapted from [24]).

If it is presumed that the polymeric solution is sufficiently dilute, the virial coefficients bigger than the second virial coefficient *A*_2_ will be omitted in the subsequent evaluation of light scattering data. This parameter reveals the intensity of interactions between the solvent and the dissolved polymer. The positive values of *A*_2_ are characteristic of the thermodynamically “good” solvents (when polymer–solvent interactions dominate polymer–polymer ones). The slightly negative values of *A*_2_ indicate the tendency of the polymer to precipitate from the solution as the polymer–polymer interactions are stronger than those between the polymer and solvent. In the case of “theta” solvents, the *A*_2_ coefficient is zero. The structural-thermodynamic parameters of the studied system can be accurate only if the distance between scattering particles is large enough to avoid wave interference from different scattering particles (external interference). In this respect, the preparation of sufficiently diluted solutions is recommended, meaning the extrapolation of the experimental results to *c* = 0 and *θ* = 0°. The analysis of classical light scattering experiments applied to polymer solutions involves rewriting the Zimm equation in the following form:

*K* × *c*/*R_θ_* = 1/*M_w_* + 2*A*_2_ + 16*π*^2^ *R_g_*^2^ sin^2^(*θ/*2)/3*λ*_0_^2^*M_w_* (12)

with *K* as in Equation (2). Data for all *θ* angles are extrapolated to *θ* = 0° for each *c* concentration value, and data for each *θ* scattering angle are extrapolated to concentration *c* = 0 (Figure 1).

**Figure 2:**
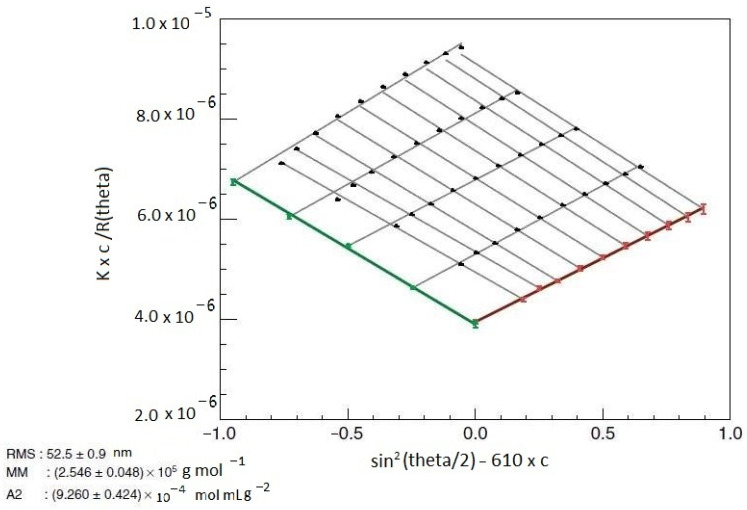
Zimm plot for a polymer with high molecular mass [26].

Bruno H. Zimm introduced a graphical representation of the extrapolations of the scattering equation by representing K*c/R_θ_ versus sin^2^*(θ/*2*)* for each measured point (*θ*, *c*). Both sets of extrapolated values correspond to two limiting curves which show and confine data from up to down, and from right to left. Extrapolations to angle *θ* = 0° stretch to the left of the abscissa, and extrapolations to *c* = 0 to the right of the ordinate. Thus, the representation is shown as a fan or grid (Figure 2).

The determination of *M_w_*, *A*_2_, and *R_g_* requires the extrapolation of experimental data to *c* = 0 and *θ* = 0° using the Zimm method and the representation of values K*c/*R_θ_* versus sin^2^ (*θ*/2) + *ac*, where *a* is an arbitrary constant chosen to obtain a clear plot. If some uncertainties regarding the values of parameters of interest appear (e.g., too high *R_g_* or large *M_w_*), Debye and Berry representations of the experimental data are used in practice.

### 2.2. Dynamic Light Scattering

**Figure 3:**
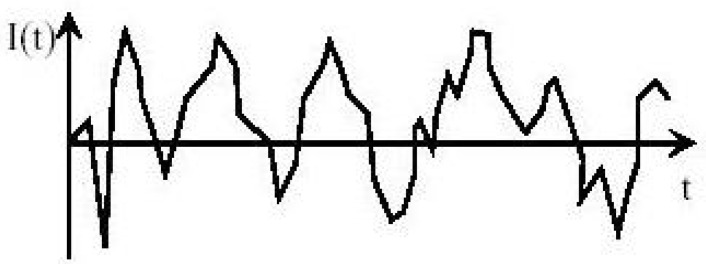
Fluctuations of light intensities determined by the random motion of particles in solution.

Dynamic/quasielastic light scattering (DLS/QELS) occurs at the same wavelength as the incident light, and the fluctuations in the intensity of the scattered light in time are studied instead of the time-averaged intensity of the scattered light. Due to the Brownian motion of particles, the intensity of the scattered light records fluctuations in very short time intervals (Figure 3). Information about scattering particles under Brownian motion can be obtained through the mathematical analysis of the autocorrelation functions for the scattered light intensity.

Generally, autocorrelation means to correlate a signal with a delayed copy of itself as a function of delay. The dependence of the autocorrelation signal/intensity on the temporal delay is expressed by the equation:

*I_ac_*(*τ*) = ∫*P*(*t*)*P*(*t* + *τ*) d*t* (13)

where *P*(*t*) is the pulse of light at *t* time, and *P*(*t + τ*) is the delayed pulse of light.

The autocorrelation function expresses the probability that, after a certain delay time *τ*, the intensity of scattered light is identical to that from the initial time moment, so the normalized intensity autocorrelation function is defined as:

*g*(*τ*) = <*I*(*t*) *I*(*t + τ*)>/<*I*(*t*)>^2^ (14)

where *I*(*t*) is the detected/measured intensity as a function of time *t*, <*I*(*t*)>^2^ is the average scattered intensity squared, *τ* is a delay time, and the brackets represent the averaging over all *t* values. For a monodisperse sample, the normalized autocorrelation function or intensity–intensity time autocorrelation function has an exponential shape:

*g*(*τ*) = 1 + *β* e^−2*qDqτ*^ (15)

where *q* = 4π sin(*θ/*2) *λ*^−1^, *D* is the translational diffusion coefficient, *λ* is the wavelength of incident light in a particular solvent, *β* is the correlation function amplitude or spatial coherence factor, and *θ* is the diffusion angle. The analysis of the autocorrelation function generally allows the determination of the particle diffusion coefficient (particularly, of macromolecules). Using the diffusion coefficient, and assuming that the molecules have a spherical shape, the hydrodynamic radius *R_h_* can be calculated through the Stokes–Einstein relation:

*R_h_* = *k_B_T*/6π*ηD* (16)

where *k_B_* is the Boltzman constant, *T* is the absolute temperature, and *η* is the solvent viscosity. The hydrodynamic radius *R_h_* represents the radius of a sphere hydrodynamically equivalent to the investigated molecules/particles with the same *D* value as the equivalent sphere [27]. Even though DLS is a non-invasive method useful to measure the size of particles undergoing a Brownian motion in a dispersion medium, the classical Stokes–Einstein relation could be applied only to hypothetically spherical solid particles, because the mathematical relation relates the translational diffusion coefficient to the particle diameter [18]. Experimentally, DLS reports the size distributions of sufficiently largely different-size particles in diluted solutions by measuring the Stokes–Einstein diffusion coefficient. Also, DLS yields information on the complex internal dynamics of different systems like solid spheres, flexible macromolecular chains, branched polymers, or rod-like colloidal particles [28,29]. For example, the physical properties of rod-shaped nanocrystals with *L* length and *d* diameter could be determined and predicted using DLS measurements. Depending on the geometrical configuration of the polarizers that precede and follow after the sample, the light scattering measurements could be realized in two modes: vertical–vertical (V-V) mode using polarized DLS and vertical–horizontal (V-H) mode using depolarized DLS. The first mode of operation or data acquisition provides the translational diffusion coefficient *D_t_*. The second one generates the rotational diffusion coefficient *D_r_* of a molecule in solution. Practically, *D_t_* is determined from the slope of V-V and V-H mode, and *D_r_* is extracted from the intercept of V-H mode, respectively [30,31]. Then, coefficients *D_r_* and *D_t_* are related with 1/*L* and 1/*L*^3^, respectively, according to the following relations.

Rewriting the Stokes–Einstein relation (Equation (16)), *D_t_* for non-interacting spherical particles of radius *r* through a liquid is expressed as:

*D_t_ = k_B_T/6πηr* (17)

where *r* is the equivalent of *R_h_* from DLS measurements. In addition, *D_r_* for spherical-shaped particles is extracted from the Einstein–Smoluchowski equation [32]:

*D_r_ = k_B_T/8πηr^3^* (18)

In the case of rod-like or cylinder-shaped particles, the translational and rotational diffusion processes are described by corresponding coefficients as a result of the following relations proposed by Broersma [33,34]:

*D_t_* = (*k_B_T*/3*πηL*) (*δ* − *γ_║_*/2 − *γ*_⊥_/2) (19)

*D_r_* = (3 *k_B_T*/*πηL*^3^) (*δ* − *ρ*) (20)

where

*γ_║_* = 0.807 + 0.15/*δ* + 13.5/*δ*^2^ − 37/*δ*^3^ + 22/*δ*^4^ (21)

*γ*_⊥_ = −0.193 + 0.15/*δ* + 8.1/*δ*^2^ − 18/*δ*^3^ + 9/*δ*^4^ (22)

*δ* = ln(2*L/d*) (23)

*ρ* = 1.14 + 0.2/*δ* + 16/*δ*^2^ − 63/*δ*^3^ + 62/*δ*^4^ (24)

and *L* and *d* are the length and diameter of the particle. Also, it was supposed that the diffusion of a rod-like particle is composed of a faster and a slower diffusion process parallel and perpendicular to the rod axis, respectively.

Torre and Bloomfield proposed alternative equations for rods with Brownian motion and a finite aspect ratio *p*:

*D_t_* = (*k_B_T*/3*πηL*) (*δ* + 0.312 + 0.565/*p* + 0.1/*p*^2^) (25)

*D_r_* = (3 *k_B_T*/*πηL*^3^) (*δ* − 0.662 + 0.91/*p* − 0.05/*p*^2^) (26)

where *p* = *L/d* [35].

The mutual relation between *R_g_* and *R_h_* depends on the particle shape. The shape factor *R_g_*/*R_h_* is an important instrument of the scattering particle topology, especially of small particles with a size of 10–100 nm. For example, the shape factor in the case of a compact sphere could be calculated from the following equation:

*R_g_*^2^ = 3 *R_h_*^2^/5 (27)

The theoretical values of shape factor for different topologies of particles are related to their morphologies as follows: 1 for a hollow sphere; 0.775 for a homogeneous or compact sphere; 1.505 for a random coil; 0.775 ÷ 4 for an ellipsoid; and (1/3^1/2^) ln(*L*/*d* − 0.5) for a cylinder with length *L* and diameter *d* [27,28].

## 3. Laser Light Scattering Instrumentation and Detectors

The evaporative light scattering detector ELSD is an alternative to the refractive index RI and ultraviolet UV detector for the liquid chromatography analysis of substances that do not necessarily contain a chromophore group and low volatile or non-volatile compounds. Although some authors consider it to be a universal detector, ELSD cannot detect highly volatile compounds. In a chromatographic system, ELSD can operate in isocratic or multiple solvent gradient mode [36]. Within an ELSD, three processes take place successively: (I) the nebulization or atomization of the eluent or mobile phase composed of the analyte dissolved in a suitable solvent, that comes out of the chromatographic column; (II) the evaporation in a heated drift tube of the solvent from the droplets of different sizes previously formed; and (III) the detection by a photomultiplier or a photodiode of the light beam coming from a laser source and subsequently scattered by the residual non-volatile particles, namely, the analytes. The intensity of the scattered light is proportional to the concentration of the eluting particles. The performance of ELSD can be optimized by adjusting the following parameters: the nebulizing gas flow rate; the temperature of the drift tube or evaporator; and the composition of the mobile phase [37]. Even though the laser light scattering phenomenon is found only in a stage of this type of particle detection, the ELSD is often confused with light scattering detectors [27].

Being non-volatile compounds, the macromolecules can be studied using classic chromatographic methods with included ELSD. Along with the development of instrumentation that uses light scattering phenomenon, too few researchers have used chromatographic techniques with an included ELSD for the characterization of cellulosic materials. For example, the molar mass characterization of sodium carboxymethyl cellulose NaCMC was performed by Shakun M. and his team, using a size exclusion chromatography-multiangle laser light scattering (SEC-MALLS) system that also contained an ELSD [38]. Other authors developed a highly sensitive liquid chromatography method with ELSD working in gradient elution mode for the quantification in various pharmaceutical formulations of hydroxypropyl methylcellulose acetate succinate (HPMCAS), known as hypromellose acetate succinate [39,40]. From a variety of mobile phase systems suitable for ELSD detection, the authors choose the aqueous NaCl and aqueous ammonium acetate NH_4_OAc solvent systems for the characterization of NaCMC, and a water/acetonitrile mixture with formic acid was added for HPMCAS.

The goniometer is an instrument from the old generation of laser light scattering instruments composed of a glass or quartz cell containing the analyte solution, and a photodiode that moves successively to make measurements at each pre-set scattering angle *θ*. Because measuring the angular intensity dependence requires a long time for each *θ* angle, this instrument cannot be used in online configuration with other detectors suitable for the analytical separation of (macro)molecules. The instrument manufacturers have designed goniometers that allow using both SLS and DLS measurements [27].

Later, the laser photometers were designed in various experimental versions that operated at a single, two, or multiple angles. The single-angle photometers use *θ* = 90° for measurements in right-angle laser light scattering (RALLS) detection, *θ* < 90° in low-angle laser light scattering (LALLS) detection, and *θ* > 90° in wide-angle laser light scattering (WALLS) detection (usually 173° in DLS measurements). The multiangle laser light scattering (MALLS) photometers cover the whole range of *θ* at once (usually 18 positions for 18 photodiodes). The following light scattering detectors could be a part of the online SEC system: MALLS detector, LALLS detector, and RALLS detector, along with a viscometric VISC detector and a refractive index RI or differential refractive index dRI detector. Also, a MALLS photometer allows the analysis of unfractionated samples in batch measurements [27].

**Figure 4:**
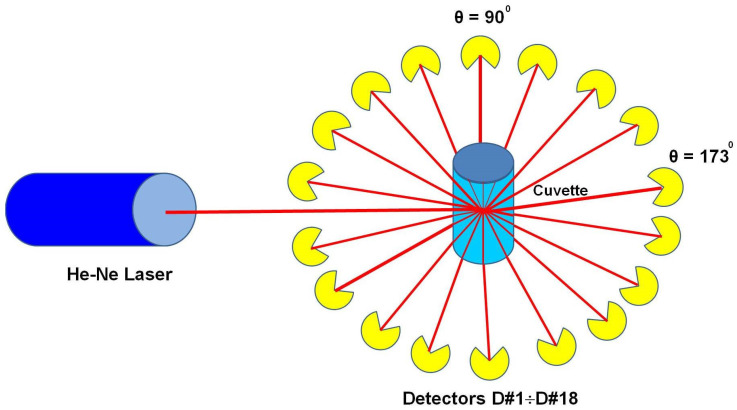
Experimental setup of MALLS experiments in batch mode with scintillation vial.

Unlike the pioneering devices that included a scanning goniometer, nowadays, these are equipped with an array of photodetectors concentrically arranged around a scintillation vial/cuvette/flow cell containing the sample. Thus, the registration of the scattered intensities under multiple angles (multiangle laser light scattering (MALLS) measurements) is allowed (Figure 4).

Since each detector can cover a solid angle different from the scattering central volume of the scintillation vial, and each detector has an amplification factor slightly different from other detectors, it is necessary to normalize their signals. Values of *I*(*θ*) sin*θ* product (where *I*(*θ*) is the intensity of the signal recorded by the device to *θ* angle) for all scattering angles are adjusted to be equal with the characteristic value measured at *θ* = 90°.

The calibration of the light scattering apparatus with toluene, benzene, or decalin, and detector normalization with an isotropic material (polystyrene, dextran, or bovine serum albumin in suitable solvents) are the basic requirements necessary to obtain experimental results as real as possible. Alongside these conditions, others are important, too: the rigorous purification (by filtration) of samples and solvents, the use of perfectly cleaned laboratory glassware, a working environment as far as possible free of dust, solution degassing (by it being simply stationary for a few days in scintillation vials, through ultrasonication, or through the application of freeze–thaw cycles), the knowledge or experimental determination of the increment refractive index *dn/dc* for the tested solution, and the counteraction of the molecular associations/aggregations in solution using low molecular salts. All these requirements will contribute to accurate experimental results generated by recordings made by many detectors, exempting those who provide noisy signals.

## 4. Data Analysis of Light Scattering Signals

A series of formalisms were developed to analyze the scattering of particles in solution and determine the above-named physical characteristics of particles.

SLS data processing involves the selection of an optimal method for the representation of experimental data based on Zimm, Debye, or Berry formalism (Equations (28)–(30)):

*K*×*c*/*R_θ_* = f (sin^2^ (*θ*/2) + a×*c*) (28)

*R_θ_*_/_*K*×*c* = f (sin^2^ (*θ*/2) + a×*c*) (29)

(*K*×*c*/*R_θ_*)^0.5^ = f (sin^2^ (*θ*/2) + a×*c*) (30)

A Zimm plot is built using a double extrapolation of multiple angle and concentration measurements to zero *θ* angle and zero concentration.

The Guinier and Kratky formalisms are other approaches to represent the experimental data:

*R_θ_* = f (sin^2^ (*θ*/2) + a×*c*) (31)

sin^2^ (*θ*/2) *R_θ_* = f (sin (*θ*/2) + a×*c*) (32)

for measurements made at low angle and infinite dilution since *P*(*θ*) = 1 [27].

The most widely used method to analyze the light scattering data is the Zimm method. The Zimm formalism gives better results for molecules with *R_g_* ~ 20–50 nm and requires a lower fit degree compared with the Debye formalism for molecules with the same size. Instead, for large molecules, the data extrapolation often produces negative values for *M_w_*.

The Debye method uses Debye formalism, and it is used over a wider range of *M_w_*, being suitable even for molecules with *M_w_* greater than ~10^6^ g/mol or *R_g_* ~ 100 nm. Due to the very large curvature of the Zimm plot, it is necessary to delete data from some high *θ* angle detectors to improve the fit of the extrapolation.

If the Zimm experimental data processing method fails for large molecules, it is recommended to use the Berry formalism in combination with discarding data related to large *θ* angles.

For molecules with *R_g_* less than ~20 nm, all of the above-mentioned methods will give almost identical results. In the case of a polydisperse sample, it is necessary to test different data sets using all suitable methods and, finally, to report the best matching results with the minimum errors [41].

**Figure 5:**
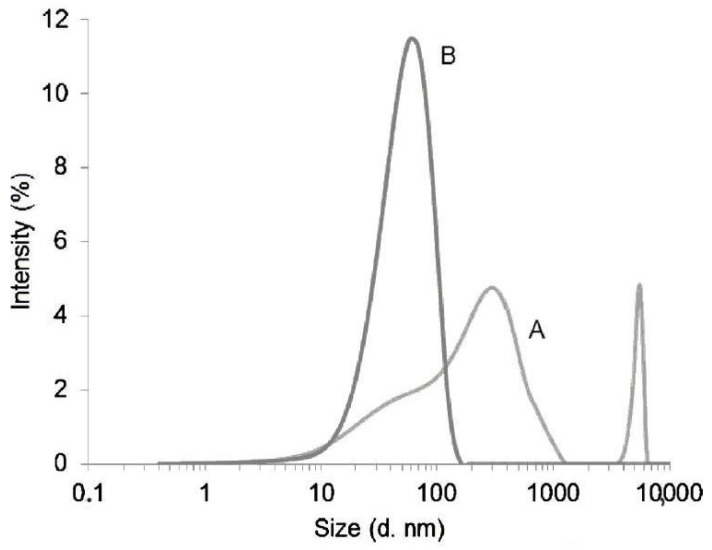
Intensity distributions obtained by means of DLS for a polydisperse sample (A) and a monodisperse sample (B) [42].

DLS data are collected using a laser light scattering spectrometer composed of a laser and a multi-*τ* digital time correlator that measures the ultrafast laser pulses. The measured photocurrent is then transformed into signals by means of CONTIN analysis and translated into monomodal or multimodal distributions of sizes for particles from a solution. Depending on the sample polydispersity, the size distribution can result as in Figure 5. 

## 5. Detector-Coupling Techniques That Include a Light Scattering Detector

SLS and DLS methods are currently used to characterize cellulosic materials. SLS and DLS apply the same phenomenon, but the difference lies in the collection and processing of the experimental data. Some experimental devices incorporate both methods and thus provide more complete information about structural-thermodynamic parameters.

**Figure 6:**
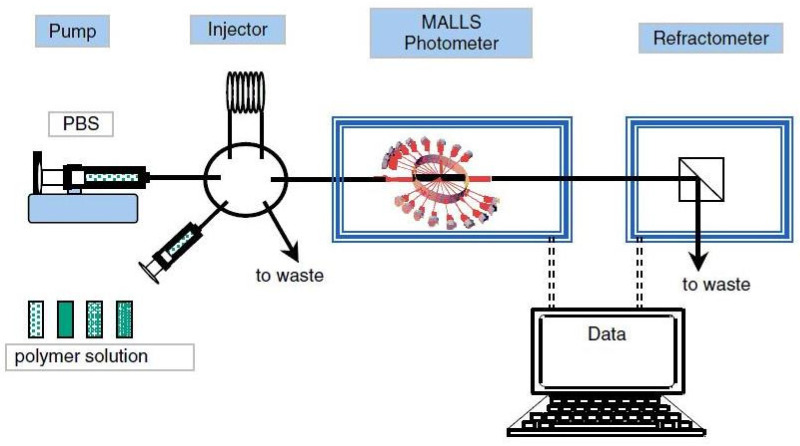
Configuration of a chromatographic system in SEC-MALLS-RI detection [26].

Detector-coupling techniques that include a light scattering detector can have various experimental configurations to realize the analytical separation of macro(molecules). Cellulose-based materials are characterized using the following technical settings: SLS-DLS detection; SEC-MALLS detection; SEC-MALLS-RI/dRI detection (Figure 6); SEC-MALLS-dRI-VISC detection; SEC-MALLS-FFFF detection; A4F-MALLS detection; AF4-MALLS-dRI detection; and SEC-MALLS-FL-RI detection, where FFFF refers to flow field flow fractionation, and A4FF is the asymmetrical flow field fractionation. An ultraviolet UV or photodiode array PDA-type concentration detector is introduced into the chromatographic system to analyze chromophore substances. A fluorescence FL detector is used if the respective samples emit fluorescent radiation [43,44]. Sometimes, researchers are interested in comparing the viscosity-average molecular weight *M_v_* value with the *M_w_* value provided by the laser photometer, in which case a VISC detector is added to the experimental setup [43,45]. To enhance the accuracy of size analysis for some samples with multimodal size distributions, an FFFF or AF4 detector is recommended to separate the particles based on their diffusion coefficient in an electric field [18,46,47].

The difference between the refractive index of the solvent and that of the polymer solution (*dn*) is measured using a refractometer. The variation of this quantity as a function of solution concentration represents the increment in the refractive index (*dn/dc*). This parameter is often required in light scattering measurements and measured with a dRI detector/differential refractometer. *dn/dc* is defined by the following equation:

*dn/dc ≈ (n_p_ − n_s_)/ρ_p_* (33)

where *n_p_* and *n_s_* are the refractive index of the polymer and solvent, respectively, and *ρ_p_* is the density of the polymer in solution. The *dn*/*dc* is specific to a particular solvent–polymer system and depends on the type of solvent, temperature, the concentration of polymer solution, the molecular weight of the polymer, and the wavelength of the light passing through the refractometric cell. The measurements of this parameter become complicated in the case of a multi-component system. For example, in the case of a bicomponent system, *dn/dc* is determined according to the relationship:

*dn/dc = w_1_ (dn/dc)_1_ + w_2_ (dn/dc)_2_* (34)

where *w*_1_ = *M*_1_/(*M*_1_ + *M*_2_) and *w*_2_ = *M*_2_/(*M*_1_ + *M*_2_), while *M*_1_ and *M*_2_ are the molar masses of the components of the mixture [27].

## 6. Molecular Weights, Particle Size, Molecular Weight Distributions, Particle Size Distributions, and Conformation for Cellulose-Based Materials in Various Solvents

### 6.1. Celluloses

Highly reactive groups of cellulose, able to establish strong and numerous intramolecular or intermolecular hydrogen bonds, represent a challenge in the dissolution of this biopolymer. Knowing that the cellulose chains are difficult to disperse at the molecular level in some solvents, different systems composed of a solvent and low-molecular-weight salt are tested. The purpose of this treatment is to counteract the hydrogen bonds and favor the interaction between cellulosic hydroxyl groups and solvent molecules. In this way, a minimum degradation and sufficient sample stability are recorded. The sample integrity will be reflected in the accuracy of the obtained physicochemical parameters. In addition, the cellulose chains are stiff and close-packed, and an extended conformation of macromolecular chains in solution is a mandatory condition to obtain realistic values of the physicochemical characteristics of the polymer.

Besides strong intermolecular interactions between cellulose chains, the appearance of the glucopyranose ring sensitive to hydrolysis and oxidation will narrow the palette of suitable solvents for cellulose. The dipolar aprotic solvents like DMAc, DMF, and *N*-methylpyrrolidone only swell the cellulose, but adding a small quantity of low-molecular-weight salts like LiCl will dissolve the cellulose. For example, according to Dibrova and Khanchich, in the case of the dissolution of cellulose in dimethylacetamide DMAc, two types of complexes can form between cellulose, low-molecular-weight salt, and solvent [20]. After dissolution, the cellulose solutions in DMAc with the addition of LiCl are stable up to 100 °C for a long time.

The ionic liquids are novel solvents of cellulose, and the following systems were tested at 50 °C: 1-ethyl-3-methylimidazolium acetate EMIMAc, and 1-allyl-3-methylimidazolium chloride [AMIM][Cl]. To enhance the chance of success, some authors tried to dissolve the cellulose in a binary solvent mixture like [AMIM][Cl]-DMSO; DMF-EMIMAc.

Jiang et al. [48] reviewed the dissolution mechanism of cellulose at low temperatures using aqueous solutions of urea or thiourea with the addition of NaOH or LiOH. The solvent system, precooled in the range of −12 to −5 °C, was mixed with the cellulose such that an inclusion complex resulted.

SEC is a routine analysis of polydisperse biopolymers with ultrahigh molecular weights but with inaccurate results regarding the distributions and average values of the molecular masses due to a possible effect of degradation during the macromolecules elution through the chromatographic system. In such cases, bimodality, extensive tailing, and/or displacements of the elution profiles towards higher elution volumes with the increase in sample flow rate were recorded [49]. All these impediments were countered when the researchers opted for a chromatographic system with multidetectors. Because the viscosity of cellulose solutions increases abruptly with the increase in cellulose concentration, forming a gel, the preparation of dilute polymer solution with a concentration lower than 15% for laser light scattering experiments is recommended.

Usually, preparation procedures for cellulose characterization by means of SEC involve an activation step of the sample with water and DMAc, followed by dissolution in LiCl such that a final sample concentration of 0.02–25% is obtained. The chromatographic systems are composed of TSK-gel, Ultrastyragel, or PL mixed chromatographic columns and include various detectors: dRI, VISC, MALLS, and UV–Vis. They operate in a wide temperature range (20–80 °C) and are calibrated with polystyrene, pullulan, or polyisoprene standards. In time, the column calibration and sample preparation have been continuously optimized by researchers, and the repeatability of measurements has improved [50].

Berggren et al. [51] proposed improved methods for the evaluation of the molar mass distribution of the cellulosic components from Kraft pulps, namely, unbleached, oxygen-delignified, or fully bleached pulps, dissolved in LiCl/DMAc. For comparison, they used two configurations: SEC-RI and SEC-MALLS-RI. The first system was calibrated with narrow pullulan standards and used direct-standard calibration. The second system was calibrated with toluene, normalized with a narrow polystyrene standard solution, and allowed absolute measurements of numerical average molecular weight *M_n_* and *M_w_*. Using the SEC-RI method, the molecular masses of cellulose were overestimated because of structural differences between cellulosic samples and pullulan standards. Applying some correction factors, the researchers correlated the *M_w_* and *M_n_* values of the cellulose obtained by means of the SEC-RI method with the ones obtained by means of the SEC-MALLS-RI method. Another way to improve the evaluation of molecular mass distributions (MMDs) for cellulose consisted of the recalculation or expression of nominal molecular masses of pullulan standards to cellulose-equivalent molecular masses. The discrepancies between *M_w_* and *M_n_*, provided by the two experimental methods, were substantially reduced using the second approach.

The popular solvent system LiCl/DMAc [52] was used to dissolve the unaged and artificially aged pure cellulose samples, but also for their subsequent analysis by means of the SEC-MALLS-dRI method [53]. Besides the relative molar masses and radius of celluloses detected by this configuration, the authors were interested in evaluating the conformation and *dn*/*dc* of macromolecules in dilute solutions. Thus they found that celluloses adopted a random coil conformation and LiCl/DMAc is a thermodynamically good solvent for these polysaccharides. Also, the mean value of 0.077 mL/g for *dn/dc* in the case of cellulosic fibers, measured off-line with an interferometric differential refractometer, was slightly different compared with the values obtained by Saito et al. [52] with a dRI detector, but in the same solvent system: 0.094 mL/g for 2,2,6,6-tetramethylpiperidine-1-oxyl TEMPO-oxidized cellulose or 0.103 mL/g for the original linter cellulose.

To overcome the aggregation, incomplete dissolution, or detrimental degradation of celluloses by heating, some researchers replaced DMAc with 1,3-dimethyl-2-imidazolidinone DMI, in a mixture with LiCl [54]. They evaluated fresh and stored solutions of microcrystalline cellulose powder (MCC), filter paper pulp (FPP), a commercial hardwood bleached Kraft pulp (HBKP), and bacterial cellulose (BC) (from *Acetobacter xylinus* subsp. *Nonacetoxidans*). Using a SEC-MALLS-RI system, all these samples were compared from a hydrodynamic point of view with a curdlan sample. Decreasing with a maximum of 9% of *M_w_* of the cellulose samples, after six months of storage, reinforced the idea that the depolymerization of cellulose samples was minimal and sufficiently stable in LiCl/DMI. Thus, LiCl/DMI was a solvent system recommended for SEC-MALLS-RI analysis. In support of this conclusion is the absence of aggregates detected in the recorded chromatograms.

In another study, Yanagisawa and Isogai [55] endowed a chromatographic system with two laser light scattering detectors, MALLS and QELS, to study various celluloses with different degrees of polymerization in LiCl/DMAc or LiCl/DMI. They studied microcrystalline cellulose powder, filter paper pulp, acid-hydrolyzed regenerated linter cellulose and tunicate cellulose (from *Halocynthia* sp.), laboratory-cooked spruce bleached sulfite pulp, commercial softwood and hardwood bleached sulfite dissolving pulp, and commercial hardwood bleached Kraft pulp. The authors estimated the Kuhn segment lengths (*l_K_*) of 24 nm for cellulose tricarbanilate in tetrahydrofuran THF, and around 18 nm for cellulose samples and cellulose tricarbanilate in LiCl/amide, meaning that, generally, cellulose and cellulose tricarbanilate have a predominantly random coil conformation in solution.

Lojewski and the team [56] analyzed two model papers artificially aged for 5 days: PAPER-1, a bleached sulfite softwood paper containing hemicelluloses and traces of lignin, and PAPER-2, a cotton paper containing pure cellulose which did not contain hemicelluloses. The authors concluded that the SEC-MALLS method was the most appropriate one to collect information on the whole molecular mass distribution, especially at a certain depolymerization stage of cellulose.

Yamamoto et al. [57] reported a complete dissolution of holocelluloses from softwood and hardwood in 1% LiCl/DMI, after a pretreatment with ethylenediamine (EDA). This fact was confirmed by the patterns with smoothed profiles, without no shoulders or large peaks, obtained for treated holocelluloses from a chromatographic analysis in SEC-MALLS-RI-PDA configuration. Residual lignin determined the light yellow color of solutions and was detected using a PDA detector at an absorption maximum of 280 nm. Molecular weight distributions for all three types of hollocelluloses were bimodal. The low molecular fraction was composed of noncrystalline hemicelluloses and the high molecular fraction of crystalline cellulose. The slope of conformational plots of 0.35–0.39 was related to a dense conformation of hollocelluloses in 1% LiCl/DMI, most probably due to the branched structure formed on the surface of crystalline cellulose microfibrils.

The aging kinetics of two types of paper used to isolate the power transformers, namely, Thermo 70 and Insuldur, with a content of 10–20% hemicelluloses and 2–6% lignin, were analyzed by means of SEC-MALLS in DMAc/LiCl. The aging process is related to the depolymerization of cellulose [58].

Because the profile market does not commercialize standards of molecular masses for cellulose, researchers often use derivatives of cellulose like cellulose tricarbanilate, and then extrapolate the results to cellulose, taking into consideration the substitution degree in cellulose tricarbanilate, usually around 2.9. Thus, Pawcenis et al. [59] used the SEC-MALLS-RI configuration to analyze cellulose tricarbanilate in THF and then reported the molar masses of cellulose to establish the influence of cellulose branching on the aging process.

To evaluate the degradation kinetics of cellulose tricarbanilates isolated from acidic wood-containing paper and aged in artificial conditions, Kacik et al. [46] used SEC-MALLS or A4F-MALLS detection to extract and compare the absolute values of molar mass and correlation coefficients between pairwise methods. They recorded the highest values of molecular weights using the A4F-MALS method, while the SEC-MALS method provided lower values.

Ahmad W. et al. proposed statistical models to simulate the degradation of alkali cellulose and compare them with the experimental results obtained using SEC-MALLS in DMAc-LiCl solutions before and after aging. They used Kraft pulp from *Eucalyptus urograndis* and monitored the changes in the molecular weight distributions (MWDs) compared with MWDs predicted by molecular simulations. Even though the scission of cellulose by means of oxidation and hydrolysis reactions was viewed as a random process, it was found that the scission determined the shifting of MWDs toward lower values of the average molecular weight as the aging time of the samples was prolonged [60].

Hiraoki and the team [61] used SEC-MALLS analysis to characterize wood cellulose in 1% LiCl/DMI and carboxyl-methylated (TEMPO)-oxidized wood cellulose in 1% LiCl/DMAc. Polymer solution analysis revealed that the weight-average degree of polymerization (*DP_w_*) value of the original wood cellulose decreased from 3100 to 2210 during the TEMPO/NaBr/NaClO oxidation due to the partial depolymerization of cellulose chains. Also, from the MMDs of carboxyl-methylated (TEMPO)-oxidized wood cellulose, it was found that the peak of the low-molecular-mass component is represented by the water-insoluble fraction of hemicellulose-related molecules.

Ono et al. [62] succeeded in dissolving in LiCl/DMAc different samples of softwood and hardwood bleached Kraft pulps noted as SBKP and HBKP, respectively, highly crystalline native celluloses from algae, tunicates, and bacteria, and cotton lint cellulose, after previously treating them with EDA. This protocol was necessary to obtain reliable conformation plots and molecular mass parameters from the SEC–MALLS analysis.

In another study, Ono et al. [63] observed that not only did the investigation method (SEC/MALLS/dRI or off-line dRI analysis) influence the *dn*/*dc* and *M_w_* values of microfibrillated cellulose, chitin, and cellulose triacetate, but also the concentration of polymer or the low-molecular-weight salt added to the solvent (LiCl in DMAc). In the case of cellulose triacetate, the two methods gave similar values for *dn/dc*. Usually, the masses for celluloses purged through an SEC system and calculated based on *dn/dc* from the off-line RI method are overestimated, so they recommended using *dn/dc* extracted from an SEC/MALLS/dRI method.

Aono et al. [64] used SLS to analyze the chain flexibility of cotton cellulose in 8 wt.% LiCl-DMAc in various polymer concentration domains. Cellulose chains in LiCl-DMAc are negatively charged such that in the dilute region, they act like a semiflexible chain and expand due to the repulsive force between intramolecular chain units. In the semi-dilute region, they will overlap and behave like a Gaussian chain or random walk model due to the presence in the solution of the negative repulsive force between inter- and intramolecular chain units. Also, the structural properties of the cellulose sample in dilute solution were as follows: *M_w_* = 82.5 × 10^4^ g/mol, *A*_2_ = 8.55 × 10^−4^ mol mL/g^2^, and *R_g_* = 61.3 nm. By means of SLS-DLS combined measurements, the researchers studied mercerized cotton cellulose dissolved in the same solvent to characterize the structures formed in the solution. These structures were grouped into fast-mode and slow-mode components based on the scattering function of the polymer solution. The first component was attributed to the molecularly dispersed chains in solution, and the second to the aggregates with multiarms formed in solution [65].

Knowing that the limiting solubility for LiCl in absolute DMAc is about 8 wt.%, Potthast A. and the team [29] proved that the water from DMAc or the binary solvent system LiCl/DMAc, usually used to dissolve cellulose, is a crucial parameter because it induces aggregation. The presence of large particles or structured aggregates within DMAC or the solvent system DMAc/LiCl was highlighted using DLS experiments. However, the researchers observed that the filtration of cellulose solutions completely removed or permanently destroyed the aggregates, and a reaggregation after the filtration step was not recorded. The authors recommended reconsidering the solvent system used to dissolve cellulose like a ternary system composed of DMAc, LiCl, and water with specific concentrations.

Mandal and Chakrabarty [66] used the DLS technique to analyze the particle size distributions of nanocellulose isolated from sugarcane bagasse, using a general calculation model for irregular particles inserted in apparatus software. The size distribution recorded particles of 18.17 nm, 32.84 nm, 37.84 nm, and 220 nm in 0.8%, 11.5%, 87%, and 0.7% volume fractions, respectively. The dimensions in the nanometric range of most hydrolyzed particles were confirmed by those obtained from atomic force microscopy AFM studies.

Lue and Zhang [67] characterized pure cellulose solutions using DLS before dispersing multi-walled carbon nanotubes (MWNTs) in a cellulose/9.5 wt.% NaOH/4.5 wt.% thiourea aqueous system to obtain a composite and demonstrate a strong interaction between MNTWs and cellulose. They observed a bimodal size distribution of cellulose macromolecules in dilute solution, with *R_h_* values of 20 nm and 157 nm, that formed inclusion complexes (ICs) which easily aggregated as single ICs or sphere-like ICs.

The characterization of pure cellulose and its composites with graphene oxide sheets (GOSs) was realized by means of DLS in NaOH/thiourea/H_2_O. Two peaks on the DLS profile were observed for pure cellulose: one attributed to single cellulose molecular chains and another to the cellulose inclusion complexes (ICs). After the GOSs addition, the second peak recorded a higher value due to the strong hydrogen bonding interactions between cellulose and GOSs [68].

To facilitate the dissolution of cellulose in LiCl/DMAc without the perturbation of crystalline structure and bulk morphology, Ishii et al. [69] proposed the sequential immersion of polymer in water, acetone, and DMAc. This solvent exchange represented a pretreatment that increased the size of pores with radii of less than 1 nm from the cellulose structure. In this way, the solvent exchange method influenced the nanometer-scale solid structure of the polymer. The porous structure of cellulose swollen in different aqueous and nonaqueous media was indirectly investigated by means of SEC and DLS in various solvents. Thus, the molecular size of low-molecular-weight solutes like acetone, diethyl phthalate, DMAc, and DMI, and, more specifically, their radius of the equivalent sphere *R_se_*, was calculated from solvent-excluded volume *V_se_*:

*R_se_* = ((3/4π)*V_se_*)^1/3^ (35)

where *V_se_* is a volume occupied by a given molecule in which the surrounding solvent molecules cannot penetrate. Studying the elusion behavior of the solutes with *R_se_* or *R_h_* dimensions from a chromatography column filled with treated cellulose, the authors indirectly found information about the pore sizes of cellulose.

Zhao et al. [70] conducted a characterization study on Na-CMC using the SEC-RI method. Then, they used DLS to analyze the starting material MCC and NaCMC, and three pharmaceutical formulations: a commercial mixture of MCC and NaCMC dispersed in water and named Avicel RC 591, a laboratory dispersion MCC + NaCMC, and a laboratory-formulated equivalent SD (MCC + NaCMC) obtained using the spray-drying method. NaCMC, analyzed at 25 °C by means of SEC-RI, recorded in water with the addition of 0.1 M sodium nitrate and 0.001 M sodium azide, an *M_w_* of 4.5 × 10^5^ g/mol, a polydispersity index of 3.55, and *dn*/*dc* of 0.137 mL/g. The average particle size values extracted from DLS measurements decreased in order: MCC (500 nm) > MCC + NaCMC (400 nm) > SD (NaCMC + MCC) (300 nm). Also, the commercial formulation, Avicel RC 591, selected in this study as comparative material, contained 30% of particles with dimensions smaller than 100 nm. It seems that all these differences in the morphology of pharmaceutical formulations are derived from the specific manufacturing process.

Besides extremely low volatility and toxicity, ionic liquids show good solubility to cellulose, chitin, polysaccharides, silk, keratin, biopolymers, and natural polymers insoluble in conventional organic solvents or water. The polymer solubility in ionic liquids is in the early stages of investigation. Ionic liquids like 1-ethyl-3-methylimidazolium bis(trifluoromethane sulfone) imide [EMIM][NTf2], 1-butyl-3-methylimidazolium hexafluorophosphate ([BMIM][PF6], 1-allyl-3-methylimidazolium chloride [AMIM][Cl], 1-butyl-3-methylimidazolium formate [BMIM][COOH], and 1-ethyl-3-methylimidazolium acetate act based on strong Coulomb interactions combined with van der Waals interactions, cation–π interactions, or hydrogen bonds.

Some authors were interested in studying the diluted solutions of MCC with a molecular mass of 9 × 10^4^ g/mol and hydroxylpropylcellulose HPC with a molecular mass of 1 × 10^5^ g/mol and a degree of substitution lower than 3, especially dissolved in ionic liquids. They observed by means of SLS that the measured *M_w_* of MCC in 1-ethyl-3-methylimidazolium acetate was higher by one order of magnitude than the value provided by the supplier. Also, the large dispersion of particle sizes suggested that the macromolecules aggregated in the form of “soft spheres”. On the other hand, the particles of HPC in ethanol were wire-shaped [71].

Due to the slow kinetic dissolution of polymers in ionic liquids and the gel formation of phase separation phenomena, it is also difficult to directly determine the values of *dn*/*dc* using a conventional differential refractometer. Thus, some authors just calculated *dn/d*c according to Equation (33) and then introduced the *dn/dc* value in SLS measurements. The same protocol was used by Chen Y. et al. [72] to study the solubility of cotton cellulose with a degree of polydispersity (DP) of about 2400 dissolved in [AMIM][Cl] at 50 °C, and the following characteristics of polymer in solutions were found from the Zimm plot: *R_g_* = 75 nm; *A*_2_ = 2.0 × 10^−4^ mol mL/g^2^; *dn/dc* = 0.028 mL/g; and *M_w_* = 7.9 × 10^5^ g/mol. In addition, from DLS measurements, it was observed that the relaxation mode of cellulose in ionic liquid was not diffusive, so the Stokes–Einstein relation could not be applied, such that the *R_h_* value of 12 nm obtained at zero scattering angle was not representative for cellulose with a *DP* of 2400. The authors recommended treating the polymer–ionic liquid system as a ternary system formed from polymer chains, solvent molecules, and ions.

The size and shape of particles, and the concentration and type of ions in the dispersed medium and surface-bound ions influence the translational diffusion coefficient *D_t_*. Boluk and Danumah [73] measured the *D_t_* of cellulose nanocrystal particles dispersed in aqueous solutions with variable concentrations of NaCl. The *D_t_* values decreased with the increase in salt concentration until the electrostatic interactions were screened out and the particle coagulation in the presence of 30 mM NaCl was promoted. Also, the analyzed particles had a *D_t_* of 5.21 × 10^−of^ m^2^/s measured using DLS, an *L* of 271 nm from Broersma’s relation, and a *d* of 15 nm measured using SEM.

The combination of Broersma’s theoretical mathematical model and computational simulation methods like the Nelder–Mead simplex direct search algorithm resulted in an optimized simulation technique. This allowed Khouri and the team [74] to study the dynamics and dimensions of rod-like systems. Firstly, they calculated the *D_t_* and *D_r_* coefficients for cellulose nanocrystals with a definite aspect ratio *L*/*d* of 17 and obtained the value of 5.048 × 10^−12^ m^2^ s^−1^ for *D_t_* and 551.9 s^−1^ for *D_r_*. Then, they experimentally determined *D_t_* = 3.0 9 × 10^−12^ m^2^ s^−1^ and *D_r_* = 355 s^−1^ from DLS experiments for a solution of 0.2% cellulose nanocrystals in water, and obtained the average sizes of nanocrystals: *L* = 253.5 nm and *d* = 15.7 nm.

Chen and Ven [75] were interested in characterizing the sterically stabilized nanocrystalline celluloses (SNCCs) obtained after 26, 42, and 84 h periodate oxidation. These samples were examined by means of DLS, transmission electron microscopy (TEM), and viscometry (VISC). The equivalent spherical diameters of the SNCCs and particle size distributions were compared with the aspect ratio estimated based on two equations for the intrinsic viscosity of the SNCCs modeled as uncharged rigid rod-like particles, and with the aspect ratio for SNCCs calculated from TEM. DLS data collected at *θ* = 90° and 25 °C indicated that the average sizes of the SNCCs decreased with the increase in reaction time: 423.8 nm for 26 h, 298.2 nm for 42 h, and 178.9 nm for 84 h. This tendency was less prominent in the results of TEM and viscometry.

Zoppe and the team [76] presented a concept toward aqueous pathways for the functionalization of cellulose nanocrystals (CNCs) with sulfonated ligands for applications in nanomedicine. Unmodified CNCs and desulfated CNCs were characterized by means of DLS and zeta potential measurements. The *z*-average particle diameter values for sulfated CNCs in 0.01 M NaCl aqueous solutions, at pH 7 and room temperature, indicated nanometer-type particles (141 nm). Desulfated CMCs recorded averaged dimensions of ten microns (about 14,700 nm). A negative value of zeta potential of −36 mV for unmodified CNCs indicated the presence of surface anionic sulfate groups. A positive value of +24.2 mV for desulfated CNCs was caused by the excess of Na^+^ cations that produced a charge reversal of anionic sulfate groups.

Cellulose from cotton linter pulp, with a viscometric molecular weight *M_v_* = 7.3 × 10^4^ determined by the supplier at 25 °C in 4.6 wt.% LiOH/15.0 wt.% urea aqueous solution pre-cooled to −12 °C, was characterized by Qin and the team [77] using a coupled SLS/DLS detector. In this study, cellulose dispersed in a mixed solvent system (7 wt.% NaOH/12 wt.% urea aqueous solution, pre-cooled to −12 °C), which formed inclusion complexes with the components of the solvent system, was purified by means of the filtration of dilute solutions through 0.45 μm filters before laser light scattering measurements. The effect of NaOH and urea composition on the stability of cellulose and aggregation behavior was monitored by changing the NaOH concentration in 12 wt.% urea aqueous solution. Two distinct signals assigned to individual cellulose chains and aggregates were observed in the particle size distribution. Each peak area (*A_single_* and *A_aggregate_*) was proportional to the weight fraction of individual and aggregated molecular size (*ω_single_* and *ω_agg_*). Consequently, the apparent value of weight-average molecular weight (*M_w_*_,*app*_) was composed of two components: *M_single_* and *M_agg_*. The ratio between *M_single_* and *M_agg_* represented the apparent mean aggregation number *N_agg_* (Table 1).

It was observed that, as the NaOH concentration increased, the stability of inclusion complexes firstly increased and then decreased, while the addition of urea constantly improved their stability. Following optimization studies, it was found that the most stable dispersion of cellulose in the inclusion complexes was achieved at 10 °C and for a solvent system of 9 wt.% NaOH/13wt.% urea; in these experimental conditions, a proportion of single chains of 0.96 and a low molecular weight of 7.6 × 10^4^ g/mol were recorded for cellulose.

Do Nascimento et al. [78] subjected the residual cotton fabric to a series of successive chemical treatments like alkali hydrolysis, bleaching, and acidic hydrolysis to obtain a colloidal suspension which they centrifuged, dispersed, and dialyzed to extract cellulose nanowhiskers. The resulting cellulose nanocrystals with a rod-like morphology were analyzed by means of DLS. These were quite stable in aqueous media at moderate electrolyte concentration (zeta potential of 25.35 ± 1.5 mV) and recorded an average size of 235 nm and a monodisperse particle size distribution. Also, the cellulose nanowhiskers showed an aspect ratio of 12 and a degree of crystallinity of 86% according to TEM, AFM, and X-ray diffraction (XRD) determinations.

Cellulose from commercial cotton linter with an *M_v_* of 4.3 × 10^4^ g/mol and 98% sulfuric acid content was acidic-hydrolyzed to obtain negatively charged cellulose nanocrystalline (CNC) particles [79]. In an aqueous solution, these nanoparticles, with an average size of about 118 nm, were well dispersed due to the sulfate ester groups and formed a stable colloidal suspension with a zeta potential of −52.6 mV. The influence of different electrolytes on the colloidal stability of polymeric solutions was monitored by means of DLS and zeta potential measurements, and the interpretation of compared data is recorded in Table 2.

Increasing the concentration of inorganic monovalent and divalent cations resulted in the aggregation of CNC particles. As compared with the salt-free aqueous solutions and with 10 mM Na^+^ solutions, the CNC particles suspended in 2.5 mM Ca^2+^ more easily tended to form much larger aggregates, suggesting less stability in the colloidal system.

The organic low-molecular-weight anionic electrolyte favored the stability of negatively charged CNC particles in suspension. The organic high-molecular-weight electrolytes induced the aggregation of CNC particles in solution because the unstable suspension of CNC particles represented the result of the entanglements or intermolecular interactions between CNCs and polyelectrolyte.

In addition, studying the influence of pH on the colloidal stability of CNC suspension, the researchers observed that, in the pH range of 2–11, the size and zeta potential values were almost constant: about 118 nm and −45 mV, respectively.

Cellulose nanofibrils, prepared from bleached eucalyptus Kraft pulp by means of oxidative pre-treatment with NaClO/NaBr/TEMPO and the mechanical treatment of different intensities, were labeled as CNF-5p and CNF-15p, and characterized using complementary methods to estimate their dimensions [42]. After the centrifugation step, the supernatants of the CNF-5p sample were analyzed by means of DLS and AFM to evaluate the particle sizes in the nanoscale range. Combining the results from these methods, namely, the average width of 14 nm measured on the films by means of AFM (W_AFM_) and the medium hydrodynamic diameter of 56 nm measured on the supernatant by means of DLS (D_DLS_), the authors used two approaches to calculate the length (*L*) of the nanofibrils based on information about the aspect ratio.

In a first approach, the authors used Equations (36) and (37):

*D_HC_*/*D_HS_* = [(2/3)^1/3^ (*L*/*W*)^2/3^]/[ln(1/*W*) + *γ*] (36)

*γ* = 0.312 + 0.565(*W*/*L*) − 0.100(*W*/*L*)^2^ (37)

where the hydrodynamic diameter of a cylinder *D_HC_*, which has the same volume as a sphere with hydrodynamic diameter *D_HS_*, was determined by means of DLS as *D_DLS_* (*D_HC_* = *D_DLS_*). The width of air-dried fibrils *W* was measured using AFM (*W* = *W_AFM_*) and *D_HS_* = (3/2*W*^2^*L*)^1/3^. In this way, an average nanofibril length of 150 nm resulted. To apply Equation (36), the aspect ratio of the cylinder should be layered in the range of 2–20 nm, but in the present case, it was 14/56 = 0.25.

So, in a second approach, they proposed Equations (38) and (39):

π/6(*D_DLS_*)^3^ = π/4(*W_A**FM**_*)^2^*L* (38)

*L* = 2(*D_DLS_*)^3^/3(*W_A**FM**_*)^2^ (39)

where *D_DLS_* and *W_A**FM**_* have the same meaning as above. For the *L* parameter, a value of 597 nm was obtained. The authors believed that a future TEM study would provide more accurate values for the nanofibril length.

Zhang et al. [80] tested the influence of dimethylsulfoxide DMSO as cosolvent on the cellulose-solvating ability of 1-allyl-3-methylimidazolium chloride. They prepared a series of binary mixtures [AMIM][Cl]/DMSO, varying the molar fractions of DMSO (X_DMSO_) in the range of 0–0.9. The light scattering fluctuations of binary solvent mixtures, analyzed using DLS, helped to calculate the decay times based on correlation functions.

The experimental results indicated that the percolation limit of DMSO in [AMIM][Cl] was reached when X_DMSO_ increased to 0.5. This low concentration of polar aprotic solvent improved the solubility of cellulose in ionic liquid. Similar results were obtained by other authors in the case of binary solvent mixtures like 1,3-dimethyl-2-imidazolidinone/1-butyl-3-methylimidazolium chloride and dimethylacetamide/1-butyl-3-methylimidazolium chloride, when the percolation limit of cosolvent in solvent was 0.6 and 0.75, respectively [81,82].

The iridescent films based on cellulose nanocrystals (CNCs) with liquid-crystalline properties are quite brittle. Bardet and the team [83] decided to add anionic sodium poly(acrylate) (PAAS) as a dispersant and neutral water-soluble poly(ethylene glycol) (PEG) as a plasticizer to a suspension of CNCs extracted from wood pulp using sulfuric acid hydrolysis, to modulate the film coloration and flexibility. DLS was used to measure the sizes of the particles formed as a result of the interaction of CNCs with these polymers. In this study, the CNCs were rod-like nanoparticles with a 250 nm length and a 5 nm width. CNCs dispersed in deionized water, PAAS, or PEG recorded a *D_h_* of 105 nm, 102 nm, and 122 nm, respectively. Their average sizes reached the value of 856 nm in suspensions containing poly(ethylenimine) (PEI). Cationic PEI was tested to elucidate the interactions favorable to deposition on CNCs. It was observed that PEG was physically adsorbed onto CNCs by means of hydrogen bonds and steric stabilization such that the self-assembling properties of CNCs were conserved and the colloidal suspension was birefringent.

Engel et al. [84] used the SEC/MALLS/dRI system to obtain detailed information about the MWDs of celluloses subjected to enzymatic treatment. At first, the native celluloses like Avicel PH 101, α-cellulose, and Sigmacell 101 were directly dissolved in a new solvent system based on DMF and 10% EMIMAc. The quantitative study revealed the following solution properties of cellulose substrates: an *M_w_* of 2.84 × 10^4^ g/mol, 7.61 × 10^4^ g/mol, and 10.9 × 10^4^ g/mol, and a polydispersity index of 3.1, 4.7, and 3 for Avicel, Sigmacell and α-cellulose, respectively. In addition, because the signals from chromatograms constantly declined with the increase in the elution volume for all three celluloses, the authors suggested a true separation by macromolecule size through the chromatographic column. Then, the regenerated celluloses, resulting from the pretreatment of commercial celluloses with the ionic liquid EMIMAc, were hydrolyzed with cellulase. For these samples, the qualitative study tracked the changes in differential weight distributions during enzymatic hydrolysis at different times. Because the MWDs for pretreated celluloses were substantially altered compared with the untreated celluloses, it was supposed that the pretreatment of the samples with ionic liquid induced a different mode of action during the enzymatic hydrolysis of celluloses.

In another study, Rein et al. [85] demonstrated that the microcrystalline cellulose powder Avicel formed true molecular solutions in binary solvent mixtures composed of EMIMAc and an organic cosolvent, polar aprotic (dichloromethane DCM, DMF, acetonitrile, or propylene carbonate) or non-polar (CLF). They used a goniometer BI-200SM and Zimm plots for SLS measurements, and a differential refractometer BI-DNDC to extract the specific properties of the polymer in solution. Thus, for cellulose investigated in the DMF/EMIMAc mixture in the molar ratio of 9:1, the *dn/dc* was 0.061 mL/g, *R_g_* = 36 nm, *A*_2_ = 2.45 × 10^−2^ mol mL/g^2^, and *M_w_* = 5 × 10^4^ g/mol, compared to *M_w_* = 4.6 × 10^4^ g/mol according to the supplier. All results attested that this solvent mixture was thermodynamically good for cellulose such that a real dissolution of the polymer at the molecular level occurred.

In the study of Zhou and his team [86], cotton linters were the source of celluloses with viscometric molecular weights in the range of (10.3–15.9) × 10^4^ g/mol. They hydrolyzed the cellulose samples with sulfuric acid to obtain the samples I-1, I-2, and I-3 or soaked the cotton linters in cuprammonium solution to obtain the regenerated cellulose samples coded as II-1, II-2, and II-3. The unfractionated cellulose samples with an estimated polydispersity index of 1.5 were dispersed and dissolved in 6 wt.% NaOH/4 wt.% urea aqueous solution at 25 °C for laser light scattering analysis. It seems that this novel nontoxic solvent ensured the dissolution at the molecular level of the samples because a colorless transparent solution resulted. SLS experiments were performed with a MALLS photometer and the *dn*/*dc* of cellulose in this specific solvent system was estimated as 0.178 mL/g. The researchers observed that the values of *M_w_* for celluloses in 6 wt.% NaOH/4 wt.% urea aqueous solution were similar to the values of *M_v_* in cadoxen calculated using the Mark–Houwink equation. Also, the cellulose chains were semiflexible in aqueous dilute solutions and presented a more extended conformation than in cadoxen.

Guan et al. [47] prepared cellulose nanocrystals (CNCs) through the sulfuric acid hydrolysis of Avicel (microcrystalline cellulose) and cotton fabric, respectively. They used coupled detectors AF4-MALLS-dRI to enhance the accuracy of particle size determination. The MALLS detector was a GaAs laser (50 mW, *λ_o_* = 658 nm) calibrated with toluene and normalized with bovine serum albumin (BSA). The authors evaluated the size distributions of different fractions of the rod-shape CNCs using the form factor model for a rod of length *L*, the relation between *z*-average mean square radius of particle ⟨*r*^2^⟩ extracted from MALLS and *L* (Equation (6)), and the Rayleigh−Gans−Debye approximation. The length of the cellulose nanocrystals derived from microcrystalline cellulose was in the range of 80–240 nm, while for CNCs derived from cotton, it was in the interval of 100–300 nm. All lengths of CNSs were slightly higher than in TEM measurements.

Braun and the team [25] hydrolyzed cotton linter with hydrochloric or sulfuric acid to obtain cellulose nanowiskers coded HA-1 and SA-1. After dialysis, the fractionated samples were marked HA-1-D and SA-1-D. The authors obtained the average length and diameter (*x_w_* and *L_w_*) of CNWs in two ways: (1) from the theoretical form factor calculations; and (2) from the Berry plot analysis. In the first approach, they compared the experimental form factor *P*(*q*) from light scattering data to the theoretical form factor *P(θ)* over a range of scattering angles and using defined values for the length and diameter polydispersity indices (*P_L_* and *P_x_*). The length polydispersity indices *P_L_* used in theoretical calculations were 2.3 for all samples, while the diameter polydispersity indices *P_x_* were 3.0 and 2.1 for samples hydrolyzed with hydrochloric and sulfuric acid, respectively. In the case of SA-1-D and HA-1-D, the *L_w_* values used for the theoretical calculations of the form factor were 270 nm and 325 nm, respectively, while the *x_L_* values were 13 nm and 33 nm. In the second approach, the dimensions of CNWs were calculated based on the *z*-average mean square radius <*S*^2^>*_z_* and the *M*_w_ of particles, measured using Berry plots, and the same polydispersity indices *P_L_* and *P_x_* were used in the theoretical calculations. MALLS measurements allowed the researchers to obtain the parameters specific to particles in solution: an *M_w_* of 1.858 × 10^9^ g/mol and an <*S*^2^>*_z_* of 241.2 nm for HA-1-D, and an *M_w_* of 3.213 × 10^8^ g/mol and an <*r*^2^>*_z_* of 257.4 nm for SA-1-D. Then, the following values of *L_w_* and *x_L_* resulted based on Equations (9) and (10): 272 nm and 13 nm for SA-1-D, respectively, and 244 nm and 22 nm for HA-1-D. In conclusion, the dimensions of cellulose spheroidal-type nanowhiskers obtained using the two calculation approaches recorded reasonable differences of up to 25%.

Alves et al. [87] used hydrolysis in sulfuric acid to extract CNCs from MCC (Avicel PH-101 with a DP of about 260), and from another two unusual sources: CMC (*M_w_* = 7 × 10^5^ g/mol, DS = 0.9) and HPMC (Methocel K15M Premium, *M_w_* = 4.3 × 10^5^ g/mol, 19–24% methoxyl, and 7–12% hydroxypropyl). The resulting suspensions were analyzed by means of DLS. Before analysis, the samples were diluted and dispersed by means of ultrasonation in water. Even though the detected *z*-average hydrodynamic diameters of the equivalent spheres do not represent the real physical dimensions of the rod-shaped particles of CNCs, this parameter was used for comparison between the samples and the calculation of the aspect ratio of the particles (Table 3). The average widths (*d*) of CNCs were estimated using SEM. Also, the lengths of particles (*L*) estimated using SEM were higher than the average sizes using DLS.

The average particle sizes of CNCs from CMC and HPMC were smaller than CNCs from MCC. Because the various functional groups from the chemical structure of the polysaccharides induce different charge surface densities, the colloidal stability of CNCs derived from MCC was poorer than the other samples, and the particles tended to flocculate.

### 6.2. Esters of Cellulose

Cellulose acetates with low toxicity, almost non-flammability, and high biodegradability result from the acetylation reaction of one, two, or three hydroxyl groups from the monomeric unit of cellulose chain. The commercial cellulose acetates are referred as “acetone soluble acetate” or “secondary” cellulose acetate (CA), with a 2.5 averaged substitution degree. The “primary” cellulose triacetate (CTA) with a substitution degree above 2.7 is known as “chloroform cellulose acetate”.

Saake and the team [88] analyzed the association of commercial cellulose acetate samples derived from a high or low catalyst process compared to their starting pulps using a multi-detector chromatographic system composed of a light scattering detector, viscometric detector, interferometric detector, styrene-divinylbenzene copolymer columns, and various acetone grade and LiBr or water addition as solvents. The samples contained different percentages of glucose, mannose, xylose, and hemicelluloses, and a different content of sulfur, sodium, and calcium. These compounds influenced the shape of chromatograms. Thus, pre-hump I was due to strongly aggregated material and was affected by the hemicellulose content. Pre-hump II was related to a minor aggregation tendency and unaffected by the hemicellulose. Pre-hump III was characteristic only of the high catalyst sample, and pre-hump IV was specific to the low catalyst sample.

The solubility of commercial cellulose triacetate in various solvents was tested by Ono and the team [63]. This polymer was insoluble in water, but soluble in DMAc or 8.0% *w*/*w* LiCl/DMAc. In addition, they used two systems and methods to determine the *dn*/*dc* values in DMAc or 1.0% *w/v* LiCl/DMAc. The online determination of *dn*/*dc* values supposed an SEC/MALLS/RI analysis system and a deflection type RI detector. The off-line analysis used an interferometric refractometer. In all cases, with or without low-molecular-weight salt added, the *dn*/*dc* for the studied polymer recorded a value of about 0.4 mL/g due to the lack of interactions between LiCl and acetate groups.

Ramos et al. [89] dissolved microcrystalline cellulose, cotton linter, and sisal, respectively, in a LiCl-DMAc solvent system, mercerized the samples in a 20% NaOH solution, and acetylated them with acetic anhydride. They obtained cellulose acetates with different substitution degrees. The acetylation reaction efficiency, expressed as a dependence of the experimental substitution degree and the molar ratio of acetic anhydride/anhydroglucose units of the cellulose (Ac_2_O/AGU) determined by means of ^13^C NMR spectroscopy, was linearly dependent on the aggregation number (*N_agg_*). The aggregation number of cellulose aggregates in solution was determined by means of SLS measurements. Because a molecularly dispersed solution of cellulose without colloidal aggregates was difficult to obtain, the cellulosic “fringed micelles” were diluted in DMAc with LiCl added. The Zimm plots were linear and the *N_agg_* resulted by dividing the *M_w_* with (*DP_v_* × 1.62) quantity. The viscosity-based degrees of polymerization (*DP_v_*) were 642, 400, and 150, and the aggregation numbers were 21, 40, and 11 for sisal cellulose, untreated cotton linter, and microcrystalline cellulose. Also, an increase in the dimensions and molar mass of the “fringed micelles” was observed with increasing cellulosic material concentration. In conclusion, the reaction efficiency was qualitatively correlated with the aggregation number of dissolved cellulose.

A series of cellulose acetates with substitution degrees between 1.72 and 2.92, obtained from industrial sources by means of saponification with NaOH, and subsequently dissolved in LiCl/DMAc, were characterized using the SEC-MALLS method [90]. The calibration curve using poly(methacrylate) standards provided overestimated values for molecular masses of cellulose acetates by a factor of about 3. Thus, the authors used these cellulose acetates with high and variable DS to construct a calibration curve and calculate the correction factors. Corrected values of molecular weights will be obtained for other cellulose acetates analyzed only using the SEC method, without a laser light scattering detector.

Most studies on cellulose acetates have been performed in polar solvents, possibly at room temperature or positive temperatures. There is little information on studies in organic solvents. Tsunashima et al. [91] tried to explain the solubility of cellulose triacetate in methyl acetate formed at negative temperatures. They analyzed the samples using DLS in two temperature regimes: 15–45 °C using a temperature-controlled bath, and from 10 °C to −100 °C using a cryostat. Besides molecularly dissolved cellulose triacetate, three types of self-assemblies were recorded at specific temperatures: 30 °C, −10 °C, and 75 °C. These self-assemblies were stabilized by keeping a balance between the intermolecular hydrogen bonds and dipole interactions. Finally, these assemblies were reorganized at −99 °C in a physical gel structure trapped in the frozen solvent.

### 6.3. Co-Esters of Cellulose

Some acetate groups of cellulose acetates could be replaced through chemical synthesis with propionate, phthalate, butyrate, or nicotinate groups in variable proportions. The new cellulose derivatives are substances soluble in simple solvents or mixtures of solvents.

Cellulose acetate phthalate (CAP) is a negatively charged cellulose derivative used as a coating material for the oral delivery of drugs, which resists gastric pH, but dissolves in the intestine. Until recently, pharmacists were only interested in the solution viscosity of this compound. Recently, Porch and the team [92] conducted molecular weight determination studies for CAP in a solvent mixture. They used a combination of column packing (wide-pore silica-based diol) and mobile phase (acetone/water/LiCl), and an online RI-LALLS detector arrangement to perform a correct SEC separation and MWD analysis of CAP.

Analyzing the molecular weights and polydispersity indices of both samples provided by Wako and Eastman, respectively, a slight difference between values was observed regardless of LiCl content, probably due to the slightly different content of phthalate groups (Table 4).

Cellulose acetate butyrate (CAB) is a co-ester of cellulose composed of a mixture of acetate and butyrate substituted hydroxyl groups. This amorphous and transparent thermoplastic has **lower moisture absorption** and better weathering resistance than cellulose acetate or cellulose propionate, and is recommended as an additive or binder in coating applications for a variety of substrates like wood, metal, plastics, and textiles. In the case of CAB with variable composition, specifically with a substitution degree for acetate of 0.8–2.1 and a degree of substitution for butyrate lying between 0.7 and 2.35, the recommended solvents are dioxane, acetone, chloroform, nitroethane, and tetrachloroethane according to ASTM International [93]. Grigoras and Olaru [94] tested the solubility of this cellulose derivative in a mixture of solvents. They correlated the conformational data extracted from off-line MALLS and dRI measurements with the composition of a solvent system consisting of 2-methoxyethanol and DMF. They found that the most extended conformation of macromolecular chains and, consequently, the higher solubility of polymer were recorded in a solvent system with a predominant content of protic polar solvent.

Another co-ester of cellulose, resulting from the esterification of cellulose acetate with nicotinic acid in the presence of pyridine and 4-toluenesulfonyl chloride, is cellulose acetate nicotinate (CAN), a polymer with a proven absorption capacity of dyes [95]. Grigoras and Grigoras [96] analyzed the solutions of CAN in DMF using the laser light scattering method to compare the molecular weight of the polymer with that of its precursor. Because the dimensions of macromolecules in solutions were smaller than the twentieth part of the value of the incident wavelength, and the samples were considered isotropic scatters, the analysis of data was performed using RALLS and Berry formalism. The ratio of molecular weights determined using laser light scattering was almost the same as that determined using SEC. This ratio was correlated to the degree of substitution of acetate groups with nicotinate moieties from nuclear magnetic resonance analysis.

### 6.4. Alkyl Esters of Cellulose

Obtaining nanoparticles with desired properties from natural polymers based on nanoprecipitation is an insufficiently exploited field. Researchers have set out to study how cellulose derivatives’ chemical composition and pendant structures influence the surface hydrophobicity, dimensions, and crystalline structure of the nanoparticles.

Zhang and the team [97] synthesized a series of cellulose alkyl esters with different pendant alkyl chains and substitution degrees: cellulose stearoyl ester (CSE 0.3; 1.3; 3), cellulose lauroyl ester (CLE 3), and cellulose caproyl ester (CCE 3). To elucidate the progress of the nanoprecipitation process of nanoparticles, the authors used solutions of the cellulose long-chain esters in THF or DMSO and precipitated in water. Then, they removed the organic solvent to obtain a suspension of nanoparticles. Information about the average size, surface charge, and polarity of the resulting particles was obtained from temperature-modulated DLS in water. In the case of the cellulose stearoyl esters, the average diameter of nanoparticles directly proportionally increased with the substitution degree from 85 nm to 200 nm. Also, with a longer alkyl length, the dimensions of nanoparticles increased from 70 nm to 200 nm, but the hydrophobicity of their surface decreased. The synthesized nanoparticles were reversible-thermoresponsive. At 25 °C, their structure was a solid nanosphere with a partially crystalline region. At 75 °C, the liquid nanodroplet structure contained an amorphous region. The drying process had different effects on the nanoparticles. It induced aggregation in the case of cellulose lauroyl ester and cellulose caproyl ester, but the individuality of cellulose stearoyl esters CSE 1.3 and CSE 3 was preserved. All these results demonstrated the versatility of cellulosic compounds as a platform for synthesizing nanoparticles with preset properties from natural polymers.

### 6.5. Ethers of Cellulose

Due to their thickening and water-retention properties, some cellulose ethers like hydroxypropylmethyl cellulose (HPMC), methylhydroxyethyl cellulose (MHEC), and methylcellulose (MC) are intensively applied in the pharmaceutical, food, and construction industries. MC and HPMC form thermoreversible gels, but MHEC has flocculation behavior. Adden et al. [98] studied one sample of MC with a DS of 1.32 and other five samples with a DS between 1.83 and 1.88. The molar mass distributions of the samples, before and after enzymatic hydrolysis, were analyzed with an SEC-MALLS/RI system. The original samples recorded an *M_w_* in the range of (1.2–22) × 10^4^ g/mol. After the enzymatic attack, MC had a molecular weight of *M_w_* = (8.4–13) × 10^3^ g/mol. The resulting oligosaccharides were prepared for subsequent analysis using mass spectroscopy.

The insolubility of cellulose in hot or cold water is due to the intramolecular bonds present in the cellulose chain. Etherification reactions of the cellulosic hydroxyl groups result in cellulose ethers like hydroxyethyl cellulose (HEC), hydroxypropyl cellulose (HPC), hydroxypropylmethyl cellulose, or hypromellose (HPMC), and ethylhydroxyethyl cellulose (EHEC). Most of them are water-soluble and biocompatible, and they are integrated into many pharmaceutical formulations.

Cellulose derivatives are defoaming surfactant substances with bulking abilities and high viscosity at low concentrations, resulting from the non-homogeneous replacing of hydroxyl groups of cellulose with methyl, hydroxypropyl, and carboxylate groups, for example. During the substitution process of OH groups, a decrease in the crystallinity degree of such substances is expected, but the crystallinity of HPMC and CMC was found to be unexpectedly high. This was the reason for using HPMC with *M_w_* = 4.3 × 10^5^ g/mol, 19–24% methoxyl, and 7–12% hydroxypropyl, and CMC with *M_w_* = 7 × 10^5^ g/mol, and a degree of substitution of 0.9, as sources for the extraction of nanocrystalline cellulose with a good yield by Alves and his team [87].

Kulicke and the team [18] studied the degradation/fractionation of some water-soluble cellulose derivative chains under ultrasonication with 20 kHz, and characterized the resulting macromolecular fragments using the combined technique of SEC-MALLS-DRI. In the case of the MHEC sample, the polydispersity *M_w_*/*M_n_* decreased from 2.4 to 1.7 as the ultrasonication time increased up to 10 min. At the same time, *R_g_* decreased from 94 to 32 nm and *M_w_* from 6 × 10^5^ g/mol to 1.2 × 10^4^ g/mol, and the molar mass distribution narrowed. In the same study, they were interested in determining the percentage of cellulose derivative that was eluted through a chromatographic column. Thus, in the case of the supramolecular structures with high molecular masses, the recovery was about 83%, 72%, and 42% for MC, MHEC and CMC, respectively. A percentage greater than 90% was recorded for the same samples, but with lower molecular masses. FFF-MALLS-DRI was the experimental version used to determine the molecular masses in the case of partially aggregated systems like HPC. The cumulative distribution function of molecular masses was composed of two distinct regions belonging to single molecules and aggregated molecules. Studying the relation between *R_g_* and *M_w_* for HPC, the authors found that the fraction of polymer with high molar masses recorded a highly compact internal structure.

Goodwin et al. [99] used ultrasounds of 22.5 W to obtain polymer fractions with reduced molecular weights from the pharmaceutical grade HEC (Klucel^®^). They tested two samples with *M_w_* values given by the supplier: HPC-EF with *M_w_* = 8 × 10^4^ g/mol (from SEC) and HPC-JF with *M_w_* = 14 × 10^4^ g/mol (from SEC). The undegraded and ultrasonically degraded samples derived from HPC-EF and HPC-JF was subjected to different methods of analysis: SEC, DLS, SLS, or VISC. The light scattering instrument was equipped with a goniometer, a laser source of 633 nm, a correlator software, and a CONTIN program, all used to obtain the *R_h_*. Then, supposing that the polymers in water were polydisperse and had a random coil conformation, the authors related *R_h_* and *R_g_* using the following relation: *R_g_* = 0.205 × *R_h_*. The *M_w_* value for the polymer in water was obtained from the Debye plot and SLS formalism. Comparing the undegraded HPC-JF sample with the degraded one using ultrasonication, it was observed that *M_w_* = 169 kg/mol determined using SEC was reduced by approximately 85–90%, and the molecular weight polydispersity decreased from 4.1 to 2.2 after 24 h. In the case of HPC with the lowest molecular weight grade, meaning the undegraded and ultrasonically degraded HPC-EF samples, the polydispersity index decreased from 2.8 to 1.9, and the *M_w_* detected using SLS was reduced from 11 × 10^4^ g/mol to 5.75 × 10^4^ g/mol after a cycle of 24 h of ultrasonication. Based on these results, the authors concluded that ultrasonic degradation is a suitable method to obtain shorter macromolecular chains, with no monomers or side reactions.

Pfefferkorn and the team [100] intended to produce three molar mass series starting from a sample of MHEC with *M*_w_ = 31.8 × 10^4^ g/mol, a substitution degree of 1.3, and an *R_g_* of 67 nm. They tested three methods for this purpose: ultrasonic degradation with a frequency of 20 kHz, oxidation with hydrogen peroxide, and autoclaving at a temperature of 130 °C. The degraded samples were analyzed with an SEC/MALLS/dRI system at 25 °C in 0.1 M NaNO_3_ solution with the addition of NaN_3_ as an antibacterial. Using the experimental value of 0.135 mL/g for *dn*/*dc*, they monitored the possible changes in molar masses, particle sizes, and MMDs. Usually, the molar masses decreased up to 10 times and the *R_g_* values up to 2 times regardless of the degradation method used. Since, after oxidation or autoclaving the samples recorded a bimodal molar mass distribution, and only the ultrasonicated samples kept their monomodal distribution, the authors concluded that ultrasonic degradation is an appropriate method to obtain homologous molar mass series. Also, for MHEC ultrasonicated samples, the following relationship of interdependence resulted: *R_g_* = 0.0511 × *M*^0.56^. The coefficient of 0.56 was attributed to an extended coil conformation of macromolecules.

Porch et al. [101] analyzed the MMDs for a sample of EHEC with *M_v_* = (10–20) × 10^4^ g/mol. The broadening of mass distribution was due to a small extent to the macromolecular aggregates present in the solution, not only to the individual chains. The SEC standard method could not be applied to characterize this water-soluble polyelectrolyte because there are no molecular mass standards with a narrow distribution required for calibration, and the universal calibration failed. Consequently, the authors proposed a LALS-RI-SEC detector configuration to analyze the aqueous solutions of EHEC. They varied the concentration of the low-molecular-weight salt from the mobile phase, and the flow rate of the mobile phase through a chromatographic system to retain the aggregates in the chromatographic columns. The resulting elution profile consisted only of the non-aggregated polymer. In this way, the real value *M_w_* = 55.2 × 10^4^ g/mol was determined. This value was obtained for EHEC dissolved in 0.1 M NaCl aqueous solution at a flow rate of 0.11 mL/min,

Jiang et al. [48] used the DLS technique to elucidate the interactions between hydroxyl groups of MC (with *M_w_* = 10.5 × 10^4^ g/mol) and sodium hydroxide/urea aqueous solvent system. Thus, they observed especially individual flexible macromolecules in MC solutions prepared using the freeze–thaw method. In solutions prepared at room temperature, multiple aggregates were formed. In the first case, hydrogen bonds between polymer, NaOH, urea, and water represented the premise of the formation of inclusion complexes between all system components. In addition, from SLS and Zimm plot analysis there resulted smaller values of *M_w_* (2.81 × 10^5^ g/mol) and *R_g_* (32 nm) and a higher value of *A*_2_ (8.39 × 10^−5^ mol mL/g^2^) for MC in solution at low temperature. For MC at room temperature, *M_w_* = 23.7 × 10^5^ g/mol, *R_g_* = 133 nm, and *A*_2_ = 7.29 × 10^−5^ mol mL/g^2^ because the solvent system used at a low temperature was better for the solvation of the polymer.

HPMC in an aqueous solution is a gelling agent useful for different industries. The sample of Methocel containing 29% methoxyl groups and 8.5% hydroxyl groups was dissolved in water and heated up to 80 °C. The structure of the interpenetrating networks resulting from different concentration regions was investigated with an SLS-DLS apparatus in correlation with the oscillatory shear measurements. In dilute solutions, the polymer recorded *M_w_* = 4 × 10^5^ g/mol, *R_g_* = 70 nm, and *R_h_* = 40 nm values, and the pores of the transient network did not exceed 40 nm. In a semi-dilute regime, above a critical polymer concentration and a critical temperature of 56 °C (where microphase separation appeared), a permanent network with pores larger than 1 µm was formed [102].

To design new drug delivery systems, it is sometimes necessary to know the thermal properties of the thermo-sensitive components. Fettaka et al. [103] studied the thermal transitions of HPC, MC, different mixtures of HPC with MC, and MHPC. Firstly, they characterized the cellulose ethers solutions of HPC, MC, and MHPC in 0.1 M LiNO_3_ using the SEC/MALLS/dRI technique at room temperature and obtained the following physicochemical parameters: *M_w_* = 28 × 10^5^ g/mol, *M_n_* = 20 × 10^5^ g/mol, and *R_g_* = 132 nm for HPC; *M_w_* = 45 × 10^4^ g/mol, *M_n_* = 16 × 10^4^ g/mol, and *R_g_* = 67 nm for MC; *M_w_* = 17 × 10^5^ g/mol, *M_n_* = 9.1 × 10^5^ g/mol, and *R_g_* = 125 nm for MHPC. Afterward, they investigated the thermal behavior in a dilute regime of concentrations and determined the thermal transition temperatures of polymers in 0.5 M NaCl by means of QELS measurements. The resulting values of 34 °C for HPC, 55 °C for MHPC, and >60 °C for MC were quite close to those determined using complementary methods like optical density and fluorescence spectroscopy. In mixtures with MC, the HPC conserved its thermal transition separately from MC.

Non-ionic cellulose ethers like HEC and HPC used as thickening and stabilizing agents have various applications in the food and pharmaceutical industries. The solution behavior of HEC and HPC in water and the presence of NaCl or Na_2_SO_4_ were studied by Marcelo G. et al. with an SEC/MALLS/RI system where the laser photometer and the interferometric refractometer operated at 633 nm [104]. The *dn*/*dc*, determined off-line with a differential refractometer, recorded almost unchanged values in salted solutions compared with water: 0.123–0.124 mL/g for HPC and 0.111–0.113 mL/g for HEC. Regardless of the type and concentration of added salt, the averaged *M_w_* for HEC increased from 1.23 × 10^5^ g/mol recorded in water to 1.42–1.5 × 10^5^ g/mol values, and the polymer behaved as a random coil in theta conditions in aqueous solutions with NaCl or Na_2_SO_4_. Conversely, the addition of 0.1 M NaCl or 0.5 M NaCl to water decreased the *M_w_* values for HPC from 1.8 × 10^6^ g/mol to 1.77 × 10^6^ g/mol or 1.15 × 10^6^ g/mol. The thermodynamic quality of the eluent increased concomitantly with the low-molecular-weight salt concentration, and Na_2_SO_4_ proved to be a nonsolvent for this cellulose derivative. The addition of the salts increased the ionic strength of the eluent, thus allowing a true size-exclusion of macromolecules from the chromatographic columns, and favoring the formation of the polymeric intermolecular aggregates in small percentage.

Varying the pH values for aqueous solutions of a commercial NaCMC sample with *M_w_* = 9 × 10^4^ g/mol and DS = 0.7, Dogsa et al. [105] found important information about the dynamic behavior of a supramolecular structure. The value of 8.1 × 10^4^ g/mol for *M_w_*, verified by SLS, was close to that provided by the manufacturer. Further, SLS allowed the determination of *R_g_* for molecular structures of CMC, while DLS monitored the relaxation modes of molecules in solution at different pH values. All these aspects were recorded at a *pK_a_* of 3.65 in the case of the electrolyte-type CMC, when the polymer molecular structure had the largest *R_g_* value (154 nm) and shortest slow relaxation times, being maximally expanded. Below *pK_a_*, a decreased charge of the polymer limited its trend to expand such that *R_g_* was 108 nm, and a slow relaxation mode characteristic of entangled globules of CMC molecules was predominant in the dynamics of the sample. Above *pK_a_*, the polymer became progressively charged, the averaged *R_g_* was 116 nm, and DLS recorded two relaxation modes in dynamics: one fast-diffusive, specific to free molecules, and the other slow-diffusive, characteristic of the mesh-like polymer network. The pH-dependent molecular structure of CMC was modeled as an expanded network with multiple non-homogenous-sized voids.

Because there are no standard substances with the same chemical structure as NaCMC and known molecular masses required to build a calibration curve, the pure SEC method does not apply to this polymer. Thus, Shakun et al. [38] proposed comparing the use of SEC-MALLS-RI detection to obtain the molar mass distributions and true molar masses for NaCMC samples with a degree of substitution in the range of 0.45–1.55, originating from cotton linters (BWL) and microcrystalline cellulose (Avicel), respectively. The aqueous mobile phase containing NaCl or a vaporizable salt like ammonium acetate could be used in conventional online 2D chromatography using ELSD, and in SEC-MALLS-RI configuration. In the first case, the conventional SEC calibration was performed with standard pullulans samples. In the second experimental arrangement, the MALLS detector was calibrated with pure toluene, and the RI concentration detector was calibrated with dextran and pullulan aqueous solutions. The authors observed that the *M_w_* values for BWL samples, determined using SEC-MALLS in 100 mmol/L aqueous NaCl, were 5 times higher than those for Avicel samples. In addition, with few exceptions, the *M_w_* values for all NaCMC samples in 100 mmol/L aqueous NH_4_OAc were almost identical to those determined in the other mobile phase. The logarithmic dependence log*R_g_*~log*M^a^*, named conformation plot, resulted in a value of about 0.61 for exponent *a*, suggesting an expanded coil conformation for the NaCMC chains in solution. Using some correction factors, the pullulan-based calibration curve was converted into a NaCMC calibration curve such that the *M_w_* values determined using SEC approached the *M_w_* values determined using SEC-MALLS.

Interactions of water-soluble cellulose derivatives with anionic or cationic surfactants, silica, and inorganic metals like Au or Ag were monitored using laser light scattering methods. In these cases, the changes in the physicochemical parameters of polymers were indirectly related to the appearance of the interactions between the polymer and the low-molecular-weight partner.

The non-ionic cellulose derivatives tend to interact with sodium dodecyl sulfate (SDS) by means of cooperative hydrophobic interactions and form complexes. The evaluation of interactions between SDS and HPC, HPMC, and HEC, respectively, in the presence of 10mM NaCl, was performed by Wittgren et al. [106] using SEC-MALLS-RI detection and Berry fitting of the experimental data. The solutions that cross the chromatographic system were considered multicomponent systems composed of the following: single polymer coils named complex of polymer; polymer adsorbed on the surfactant named polymer in the complex; and the complex itself including the first two mentioned entities. These components were formed in solutions as the SDS concentration increased to 20 mM. It seems that the substituent groups on the polymer backbone represented sites of adsorption for the surfactant. The variations of *M_w_* values for each component were evaluated. The lowest level of interaction was recorded in the case of HEC, while the highest one was found in the case of HPMC.

The interactions between the anionic SDS and hydrophobically modified ethyl (hydroxyethyl) cellulose HM-EHEC were studied by Lauten and Nystrom [107] with the DLS method at *λ*_0_ = 514.5 nm and a scattering angle of 90° in terms of the relaxation times. In the case of the uncharged polymer EHEC, the polymer–surfactant complex started to form at a critical aggregation concentration. In the case of the hydrophobically modified polymer, the binding mechanism of SDS on HM-EHEC was different. Studying the dilute aqueous mixtures of HM-EHEC with SDS, the authors observed an association phenomenon whose kinetics changed over time simultaneously with the variation in polymer and surfactant concentrations. In the absence of a surfactant, the decay of the correlation function for the dynamics of polymer chains in solution was not affected by the time evolution. A bimodal distribution of the relaxation times was recorded in the presence of a surfactant. If a small amount of surfactant was added to a very dilute HM-EHEC solution, the polymer–surfactant complexes formed by strong associations grew over time such that the huge aggregates sedimented. With the increasing in SDS concentration, the hydrophobic interpolymer associations were solubilized, and the slow relaxation mode ceased to depend on time. At higher concentrations of HM-EHEC, the polymer–surfactant complexes changed their conformation with time, and the intramolecular crosslinks were favored such that the clusters started to contract. After mixing the solutions of the two partners, the fast relaxation time was independent of time.

The aggregation of HPC as a semi-rigid polymer with amphiphilic character in the presence of variable concentrations of anionic surfactants like sodium cholate CS, sodium deoxycholate DC, and SDS was studied by de Martins and his team [108], using turbidimetry and light scattering methods, and a goniometer working at 633 nm. First, they determined the main properties of the polymer in aqueous solution from differential refractometry and SLS measurements, and the Zimm plot extrapolation of experimental data: *dn/dc* = 0.1349 mL/g, *M_w_* = 1.45 × 10^5^ g/mol, *R_g_* = 37.7 nm, and *A*_2_ = 5.21 × 10^−4^ mol mL/g^2^. DLS data evidenced the influence of the added surfactant on the dynamics of HPC. The time correlation functions, composed of a slow mode and a fast mode, and the variation in the *R_h_* values suggested that the aggregation between polymer and surfactants was carried out in two stages: the appearance of aggregation nuclei as a result of the attachment of surfactant unimers to the polymer chains, followed by the binding of surfactant micelles on these nuclei.

The same aggregation mechanism was proposed In the case of systems formed by HPMC and any of the three surfactants (CS, DC, and SDS), too. In this study, the dialyzed HPMC had *M_w_* = 3.4 × 10^5^ g/mol, *R_g_* = 71.5 nm, *R_h_* = 25.4 nm, *A*_2_ = 7.84 × 10^−4^ mol mL/g^2^, and *dn/dc* = 0.1371 mL/g [109]. Each surfactant had a different contribution to the aggregation process with the cellulose derivative. The SDS-HPMC system was more stable than bile salt–HPMC systems. The relaxation time distribution functions analyzed using DLS for polymer–surfactant systems showed a slow correlation mode and a fast correlation mode, respectively. The first one was characteristic of the interchain polymer–surfactant complexes and polymer clusters. The second one was assigned to free micelles, intrachain polymer–surfactant aggregates, or single polymer chains.

The sulfonatoalkoxy-substituted hydroxyethylcellulose is an anionic cellulose derivative obtained by Beheshti et al. [110], starting from its uncharged analog with *M_w_* = 4 × 10^5^ g/mol. The structure and dynamics of aqueous mixtures of anionic hydroxyethylcellulose HEC(−) with anionic SDS or cationic Gemini (+) were evaluated using light scattering and turbidimetry. The DLS apparatus operated at *λ*_0_ = 514.5 nm and the normalized time correlation data for a scattering angle of 75° were recorded. The experimental results suggested a weak association between the anionic HEC and SDS, but a strong interaction with Gemini(+) surfactant based on attractive electrostatic forces. In the case of the polyelectrolyte–CTAB complex, large interchain complexes appeared as the surfactant concentration increased, and the slow relaxation mode from the bimodal correlation function became dominant as the clusters grew.

### 6.6. Other Heterogeneous Cellulose Derivatives

Xanthation, cyanoethylation, periodate oxidation, and silylation are chemical reactions used by chemists to modify the structure of celluloses and obtain new derivatives with improved physical and chemical properties like cellulose xanthogenate, cyanoethyl cellulose, dialdehyde carboxymethylcellulose, and trimethylsilyl celluloses.

Hypromellose acetate succinate (HPMCAS) is a mixture of esters of HPMC with medical application. Chen et al. [111] developed an SEC method using a MALLS detector and a mixed solvent system for the direct and accurate determination of the absolute *M_w_* of HPMC and HPMCAS with a low, medium, and high acetyl/succinoyl ratio denoted as HPMCAS-LF, HPMCAS-MF, and HPMCAS-HF. According to the multitude of data regarding *M_w_*, *M_w_/M_n_*, and *R_g_*, recorded in various solvent systems, the best solvent for HPMCAS-HF was DMAC with 0.75% LiCl. For the other two HPMCAS sub-classes, the solvent system composed of 40:60%, *v/v* acetonitrile–aqueous buffer (50 mM monosodium phosphate with 0.1 M sodium nitrate) was the best.

Chemical technologists obtain a fabric called viscose by dissolving the sodium salt of cellulose xanthogenate in NaOH solution. Fisher and the team [112] used commercial Eucalyptus sulfite pulp as the starting material for viscose synthesis. They were interested in the distribution of substituents along the cellulose chains to the molecular masses for cellulose xanthogenate and amido carboxymethyl cellulose. For this purpose, they involved a multidetector system of SEC-MALLS-dRI-UV type for characterizing the cellulose ethers. Generally, the distribution of the substituents along the macromolecular chains was quite uniform such that *M_w_* distributions did not present any tails, even though the width of the MWDs of cellulose derivatives varied depending on the treatment applied to the sample in the ripening step.

Researchers used cyanoethylation to obtain ether derivatives with improved properties, especially mechanical and dielectric ones. In the case of cellulose, this chemical modification was realized in NaOH/urea or LiOH/urea aqueous solutions, with or without a derivatizing reagent. For example, Li et al. [113] tested the homogeneous cyanoethylation in LiOH/urea aqueous solutions and using acrylonitrile, in the case of two samples of cellulose, namely, absorbent cotton with *M_v_* = 2.69 × 10^5^ g/mol and cotton linter pulp with *M_v_* = 1.14 × 10^5^ g/mol. They intended to obtain cyanoethyl celluloses with different degrees of substitution and high molecular weights. The samples with DS in the range of 0.47–1.01 were soluble in water or dilute alkali solutions. The samples with increased DS dissolved in organic solvents. Using an experimental combination of SLS and DLS, the authors determined the molecular parameters and dilute solution properties characteristic of water-soluble and water-insoluble cyanoethyl celluloses. Thus, the *M_w_*, *R_g_*, *A*_2_, *R_h_*, and *R_g_*/*R_h_* ratio values were evaluated. In conclusion, in the case of the water-soluble samples, the *M_w_* was in the range of (3.51–3.93) × 10^5^ g/mol, and *A*_2_ was negative, suggesting a spontaneous aggregation in 0.9 wt.% NaCl aqueous solutions; also, the bimodal distribution of particle dimensions in DLS attested to the coexistence of the individual chains with their associations. The water-insoluble samples recorded *M_w_* values from 3.81 × 10^5^ g/mol to 4.19 × 10^5^ g/mol and showed extended stiff chains in solutions of 0.5% LiCl–DMAc [113].

The self-assembly of polysaccharides with medical applications is an insufficiently exploited subject. Starting from cotton linter pulp with *M_v_* = 11.2 × 10^4^ g/mol, Song and the team [114] designed a new synthetic route for the synthesis of an amphiphilic cellulose derivative containing 2-hydroxypropyl trimethylammonium chloride as hydrophilic quaternary ammonium groups, and hexadecyl as hydrophobic pendant long-chain alkyl groups. The hydrophobically modified quaternized cellulose formed micelles in water, and the cationic surface of the micelles facilitated the uptake of the incorporated drug to the negatively charged cell membrane. This cellulose derivative was proposed as a carrier system for poorly water-soluble drugs. The physicochemical properties of drug-free micelles and drug-loaded micelles were monitored using DLS.

The conversion of CMC to dialdehyde derivative, involving periodate oxidization in acid solutions, was investigated by Li et al. [115] for variable periodate dosage, reaction times, temperatures, and pH values. The authors demonstrated by means of laser light scattering measurements that the oxidation process occurred concomitantly with the physical and chemical degradations. The bimodal *R_h_* distributions of CMC and dialdehyde carboxymethylcelluloses with different contents of aldehyde groups were measured by means of DLS, using the scattering angle of 90°. The CMC macromolecules in an aqueous solution formed aggregates through hydrogen bonds, while in the case of oxidized CMC, the maximum for both peaks shifted from 4000 nm and 45 nm up to 83 nm and 10 nm, respectively. This fact was related to the appearance of some short fragments resulting from the disruption of the bulk dialdehyde carboxymethylcellulose during the periodate oxidation.

Tan et al. [116] intended to use dialdehyde carboxymethylcellulose as a crosslinker for collagen cryogels. For this purpose, they needed to know more precisely the size and molecular mass in the KCl aqueous solution of this cellulose derivative. Using an apparatus with SLS-DLS combined modes of working, they found the following: a *M_w_* of 2.38 × 10^5^ g/mol, a bimodal distribution of hydrodynamic radius with the main peak at 60 nm and a shoulder at about 10 nm, and a form factor *R_g_/R_h_* of about 1.3, specific to a polymer with coil conformation in a good solvent.

Demeter et al. [117] studied the solution behavior of trimethylsilyl cellulose (TMSC) derivatives prepared from microcrystalline celluloses of Avicel 101 and 102 types. The molecular parameters for cellulose derivatives with DS of 1.7–2.7 resulted from silylation and partial desilylation were obtained by means of SLS and DLS in THF at 25 °C. Thus, *M_w_* values in the range of (1.4 × 10^5^–53 × 10^6^) g/mol, *R_g_* values in the range of 150–205 nm, and *R_h_* values in the range of 550–770 nm were recorded, with few exceptions. In addition, the internal structure of the particles was studied, using the Kratky plot to determine the Kuhn segment length *l_K_*, and the Casassa–Holtzer plot to find the number of side-by-side aligned chain sections. The large values for *M_w_* and *R_g_* represented signs of macromolecular aggregation, suggesting a densely packed structure composed of bundles of 2–6 chains with *l_K_* between 120–175 nm.

## 7. Applications of Cellulosic Materials in Life Sciences

### 7.1. Wastewater Treatment

In an intensely industrialized society, the problem of the decontamination of water sources using the most ecological methods will represent a research topic until humanity understands how to protect its natural resources both for current and future generations.

Various studies have tested the ability of polymers to recover or decontaminate wastewater from mining operations. In mining, the gangue’s impurities like sand or soil are closely mixed with the ore’s minerals like talc, plagioclase, pyroxene, chromite, or chalcopyrite. The polymeric depressants like carboxymethyl cellulose, guar gum, starches, and dextrins, added to the mining gangue, will enhance the hydrophilic character of the gangue and inhibit the flotation of the valuable minerals with metallic nature. Even though the mechanism of preferential adsorption of polymeric depressants onto the charged surface of the minerals has not yet been clarified, it was supposed that it involves hydrogen bonds, electrostatic interactions, or hydrophobic interactions [118].

Talc is a naturally hydrophobic and floatable magnesium phyllosilicate, part of platinum exploitation. Parolis et al. studied the influence of metal cations, usually existing in a water plant, on the behavior of carboxymethyl celluloses as talc depressants [119]. They found that there is competition between various types of interactions. These cations interact with the negatively charged edges of the talc surface or with the anionic polysaccharides, while the carboxymethyl celluloses can inhibit the flotation of talc due to a complex between the carboxyl group and magnesium ions. The molecular masses *M_w_* of the carboxymethyl celulloses with a substitution degree in the range of 0.78–1.36 were in the range of (10.8–18.5) × 10^4^ g/mol, measured with a chromatographic triple detection system consisting of refractometric, viscometric, and dual angle light scattering detectors. Correlating the adsorption and chromatographic data, the authors observed that in the case of the polysaccharide sample with DS = 0.78 and initial *M_w_* = 15.5 × 10^4^ g/mol in the presence of talc and different electrolytes, the measured *M_w_* for the polymer varied as a function of the type of electrolyte. Thus, the *M_w_* of CMC was 113.95 × 10^3^ g/mol, and the calculated talc area surface covered by the polymer was 13.54 m^2^ in the presence of a Ca(NO_3_)_2_ solution with 10^−2^ ionic strength. In the KNO_3_ solution, the molecular mass of the polymer was 152.47 × 10^4^ g/mol and a surface of 5.38 m^2^ of talc was covered by a charged polymer. Regardless of the type of polyelectrolyte, it seems that the polymer was adsorbed in a monolayer on the surface of talc, but was more tightly coiled in the presence of divalent cations than in the case of monovalent cations. The divalent cations enhanced the adsorption of polymers on talc, promoting the depression of talc by CMC. This phenomenon was less observable in the presence of K^+^ cations.

Compared with other techniques, adsorption represents a more feasible method for pollutant decontamination from wastewater. One of these pollutants is Rhodamine B, a dye used in the food and textile industry, and a fluorescent tracer for determining water courses and flows. The researchers are in a continuous search for natural adsorbents for pollutants. Ultrafine fibers of CAN with a diameter between 400 and 700 nm, obtained through electrospinning, were tested in the presence of Rhodamine B aqueous solutions at pH 6.5. The adsorption efficiency of the adsorbent materials was related to the substitution degree of acetyl groups from cellulose acetate; it was about 74% for CAN 1.73 and 18% for CAN 2.43 [95].

### 7.2. Pharmaceutical Industry

Frequently, cellulose and its derivatives are components of antimicrobial materials, pharmaceutical formulas, and drug loading and release systems.

Smiechowicz and the team [120] prepared cellulose fibers with silver nanoparticles generated using three methods to design new materials with a wide/broad antimicrobial spectrum. They used Lyocell type cellulose as the support material, 50% aqueous solution of *N*-methylmorpholine-*N*-oxide as the solvent, silver nitrate as the substrate, propyl ester of gallic acid as an antioxidant, and light radiation as a physical agent for the generation of silver nanoparticles. Even though they varied the light conditions and exposure time, the best results were obtained when the cellulosic pulp was incubated for 24 h in a dark room. In this way, according to the DLS investigation, numerous silver nanoparticles with the smallest diameter of 12 nm and smallest aggregation of about 67 nm resulted. By incorporating silver nanoparticles, the mechanical and hygroscopic properties of the unmodified fibers were not significantly influenced.

Oxidized celluloses are a category of antimicrobial materials used as surgical cotton or wound gauze. Other oxidized celluloses play the role of drug delivery systems or broad-spectrum antimicrobial drugs. For example, spherical particles of tricarboxycelluloses, synthesized by Sharma et al. [121] from 6-carboxycelluloses, recorded antimicrobial activity comparable with the anti-tuberculosis drug Isoniazid. These particles have a narrow size range of 25–35 nm and a polydispersity index of 0.169 as the DLS analysis revealed.

Microcrystalline cellulose (Avicel, Emcocel, and Vivapur), powdered cellulose, silicified MCC, CA, and CAP are pharmaceutical excipients with the role of adsorbent, diluent, disintegrant, binder, glidant, filler, taste masking, coating, or suspending agents of food products and pharmaceutical formulations like tablets, capsules, and transdermal or intravascular drug systems. These materials are generally nontoxic and nonirritant, but when consumed excessively, they produce a laxative effect or induce the formation of granulomas in various parts of the body. The purification of the partially depolymerized cellulose results in white, odorless, and tasteless MCC with different moisture grades and particle sizes. Powdered cellulose with an average particle size in the range of 60–200 nm is commercialized under the name of Arbocel. Silicified MCC is a physical mixture of MCC particles and 2% *w*/*w* colloidal silicon dioxide. Cellulose acetate and other cellulose esters are compatible with many plasticizers and form drug-loading systems with controlled release properties [122].

Zhao et al. [70] were interested in the strength of the interactions between MCC and NaCMC because these two polymers are part of the commercial product Avicel RC 591, a hydrogel dispensed into the oral or nasal cavity. A polydispersity of 3.55 and a molar mass (*M_w_*) of 4.5 × 10^5^ g/mol for NaCMC were determined using a chromatographic system including a refractometer. The particle size distributions of MCC suspension, MCC + NaCMC laboratory system, commercial product Avicel RC 591, and dispersions of a spray-dry blend of NaCMC and MCC were determined at large scattering angle (150°) by means of DLS. These analyses, complete with a rheological study, revealed that the hydrogen bonding and ionic interactions were involved in the formation, breakup, and recovery of the hydrogel, both in its laboratory form and in commercial form.

Shukla and Tiwari [123] revised the applications of cellulose derivatives in the pharmaceutical industry. Cellulose, CAP, HPMC, and HPMCP are usually resistant to gastric fluids with acidic pH, but soluble in the mildly acidic to slightly alkaline medium of the intestine, characteristics that recommend them to be used as tablet excipients and controlled-delivery or coating components in pH-dependent oral medicaments. The dissolution pH of these polysaccharides is by their degree of substitution. When researchers designed oral delivery systems, some cellulosic derivatives were studied in combination with other polysaccharides to mutually potentiate their properties: HPMC-pectin, HPMC-NaCMC, HPMC-chitosan, and EC-starch. HPMCAS is also part of pharmaceutical excipients and coating materials [111].

Enteric prodrugs are polysaccharide–drug conjugates containing, for example, budesonide, nalidixic acid, flufenamic acid, or 5-aminosalicylic acid. Other authors proposed the use of cellulosic hydrogels for the incorporation of drugs specific to the treatment of colon diseases. CAP, EC, HEC, HPC, HPMC, and HPMCP constitute matrices for colon-specific drug delivery systems. In contrast to non-enteric esters like CA, CAB, and cellulose acetate propionate, enteric cellulose esters dissolve in different portions of the intestines following the pH of the medium and with the degree of esterification of the samples [123].

The Eudragit L100/CAB particles loaded with nifedipine were prepared by Ramesh Babu V. et al. by means of the solvent evaporation technique and using poly(vinyl alcohol) as the emulsifier [124]. Nifedipine is a photosensitive drug used to treat hypertension, while polymers are frequently used as matrices and coatings in oral formulations. The resulting microspheres were analyzed using a laser particle size analyzer and recorded mean sizes between 100 and 120 μm following the degree of drug loading. The microspheres resisted in the gastric medium up to 12 h, favoring the bioavailability of the drug.

The amphiphilic micelles of cellulose, quaternized with 2-hydroxypropyl trimethylammonium chloride and modified with hydrophobic hexadecyl moieties, were loaded with prednisone acetate, a hydrophobic anti-inflammatory drug. It was observed that after drug incorporation, the *R_h_* of micelles slightly decreased compared with the drug-free micelles, probably due to the strengthening of the hydrophobic interactions between hexadecyl moieties from the micelles’ core [114].

Rokhade et al. [125] developed semi-interpenetrating polymer network (IPN) microspheres from NaCMC and gelatin using glutaraldehyde GA as a crosslinker. Depending on the composition of each formulation, these matrices allowed the encapsulation of ketorolac tromethamine up to 67% and a controlled in vitro release of the drug according to a non-Fickian mechanism. Ketorolac tromethamine belongs to the class of non-steroidal anti-inflammatory drugs, and it is also an analgesic agent. According to DLS measurements, the IPN microspheres recorded sizes in the range of 245–535 μm.

### 7.3. Personal and Health Care Industry

Due to their biocompatibility, cellulose derivatives became attractive materials for the cosmetics industry and biomedicine. It is supposed that the presence of cellulosic surfactants will improve the practical properties of commercial products like toothpaste, soap, body wash, shampoo, washing powder, and dermatocosmetics. When they are part of skin creams, shampoos, or eye solutions, they have the role of reducing the irritation of surfactants, improving the moistening of skin and the wettability of hair, and treating and conditioning eye lenses. Complexes of cellulose nanocrystals with chitosan oligosaccharide, *β*-cyclodextrin, or fullerols can encapsulate diverse vitamins or scavenge the free radicals such that the resulting products have antioxidant effects [126]. To predict the performance of the final product and to control the manufacturing process, detailed molecular information about involved polymers is needed.

Cellulose nanocrystals, materials extracted from cellulose via acid hydrolysis, are found in cosmetic and biomedical products. The physical properties like average dimensions and diffusion coefficients of cellulose nanocrystals in solution were predicted by Khouri S. et al. and determined using DLS measurements [74].

Polymer JR trademark, also named polyquaterium-10, is a quaternary ammonium salt of HEC with anti-static and film-forming properties, included in hair, skin, or eye care products. Using SEC with triple detection (LS-dRI-VISC) and four different mobile phase compositions to counteract the effects of unwanted ionic interactions between the column packing material and the polymer molecules, Liu and the team [45] determined the absolute *M_n_* and *M_w_*, MWDs, hydrodynamic sizes, and conformation of three cationic HEC derivatives with different viscosities: Polymer JR 125, Polymer JR 400, and Polymer JR 30M. They observed that a combination of organic solvent and high buffer content considerably reduced the unusual exclusion separation phenomenon of the Polymer JR with a high molecular weight.

SLS measurements in micro-batch mode with MALLS-RI detection and Zimm plots analysis were applied to study the solution properties in PBS for Polymer JR 125, Polymer JR 400, and Polymer JR 30M. These cationic HEC derivatives recorded values of 0.139–0.146 mL/g for *dn*/*dc*, *M_w_* values in the range of (2.6–10.3) × 10^5^ g/mol, and an *R_g_* of 53–130 nm. Also, from a log-log plot of *R_g_* versus *M_w_* for Polymer JR samples, an exponent of 0.6 resulted, which is characteristic of macromolecules with expanded coil conformation [26].

### 7.4. Food-Packing Materials

In the food sector, the need for long-term and high-quality preservation of food is a necessity for the population. For this purpose, various biodegradable materials are tested, but they must also fulfill certain mechanical and oxygen barrier properties [126]. Thus, nanocomposites of CNC with poly(3-hydroxybutyrate-co-3-hydroxyvalerate) (PHVB) recorded better properties than pure PHVB films [127], and guar gum films filled with CNC were of a higher quality than pure guar gum films [128].

### 7.5. Restoration of Heritage Objects

Paper is a composite consisting of cellulose fibers from wood or cotton and different additives for filling, pigmentation, preserving, sizing, or bleaching. Papermaking is a staged process that includes the wet beating of cellulose fibers to change their morphology, the pressing of the resulting wet sheet, drying to form direct interfiber hydrogen bonds or nano-fringed fibers, finishing, printing, dyeing, or coating. Some authors tried to improve the physicochemical properties, especially the mechanical resistance of the paper. Thus, they set out to fill the gaps between the fibers and fibrils with organic and inorganic nanoparticles. The resulting nanopaper contained rod-like cellulose nanoparticles and ellipsoidal chalk nanoparticles. The size distributions of nanoparticles in aqueous suspension were studied using DLS. The hydrophilic cellulose nanoparticles recorded an average length of 200 nm, while the hydrophobic chalk nanoparticles have an average diameter in the range of 100–150 nm. Due to the new hydrogen bonds formed with the fibrillated fibers of cellulose, only the cellulose nanoparticles introduced in the pulp increased the strength of the paper and decreased its porosity [129].

Restorers face multiple challenges when studying old manuscripts and want to slow down the deterioration of aged cellulose samples, in order for them to be displayed to the public. Hanji is an ancient Korean paper, traditionally handmade in the 15th century. In those days, it was believed that treating paper with beeswax would improve its long-term stability. Jeong et al. [44] compared samples of Hanji paper, treated or not with beeswax. Besides the macroscopic differences in their qualities, the authors intended to analyze the samples at the microscopic level. They labeled the cellulose samples with carbazole-9-carboxylic acid [2-(2-aminooxyethoxy)ethoxy] amide (CCOA) and 9H-fluoren-2-yl-diazomethane (FDAM). Then, they used SEC-MALLS-FL-RI multidetector analysis in DMAc/LiCl (9%, *w*/*v*) to observe the potential changes in the MMDs, the values of *M_n_*, *M_w_*, and *M_z_*, or the polydispersity indices. Considering the natural aging process over 400 years, the untreated paper samples underwent minor hydrolysis and oxidation processes, and the molecular masses still had high values of the order (7.7–9.7) × 10^5^ g/mol. Conversely, the beeswax-treated manuscript pages were composed of cellulose with a radically decreased *M_w_* of (20.7–35.2) × 10^4^ g/mol. In the case of the beeswax-treated ancient celluloses, the degradation mechanism was pure hydrolysis. Microorganisms like *Aspergillus versicolor*, *Penicillium polonicum*, *Biscogniauxia atropunctata*, *Ceriporia lacerate*, or *Irpex lacteus* used the beeswax as a carbon source for their metabolism and secreted cellulolytic enzymes and citric acid. Based on these observations, the authors recommended preserving the beeswax-treated samples in unfavorable conditions for microorganisms that feed on beeswax to minimize the future degradation of the manuscripts.

## 8. Conclusions and Future Perspectives

- In the case of cellulose-based materials, the optimization of the final properties is closely related to the specific parameters of the macromolecules in solution: the average molecular weights *M_w_*, *M_n_*, and *M_v_*, the second virial coefficient *A*_2_, the radius of gyration *R_g_*, the hydrodynamic radius *R_h_*, the molecular weight distributions, the particle size distributions, and the conformation. These parameters are considered when the reproducibility of the polymer behavior in solution is sought.
- Determining the characteristic parameters of cellulosic compounds in solution using laser light scattering methods requires rigorous experimental conditions.
- Because no ideal GPC standards for molecular weights of cellulose-based materials are available, laser light scattering measurements are recommended in batch mode or online coupled mode as a function of the filtration grade and the polydispersity of the sample.
- Laser light scattering methods are absolute methods that provide direct information on cellulose-based materials. The main advantages of SLS and DLS are their high sensitivity, the possibility to use the same species before and after the experiment, and their compatibility with detectors like SEC, VISC, FFFF, UV-Vis, or dRI for completely characterizing macromolecules in solution. In addition, laser light scattering measurements allow the analysis of unfractionated samples in batch mode configuration. In practice, the molecular masses and MWDs from laser light scattering are compared with the values from viscometry and chromatography. Also, the *R_g_* and *R_h_* are compared with macromolecular sizes from TEM, AFM, or different theoretical models.
- A rigorous dissolution of cellulosic materials will prevent their aggregation in the solution at the molecular level and favor obtaining values as close as possible to reality for *M_w_*, *R_g_*, *R_h_*, and *A*_2_. In practice, the solvents or binary solvent systems are used at temperatures close to room temperature (aprotic dipolar solvents with a small concentration of added LiCl; ionic liquids) or below 0 °C (aqueous solutions of urea/thiourea with the addition of NaOH or LiOH). In some cases, the pretreatment of cellulose is required to dissolve cellulose in appropriate solvents. The *A*_2_ values represent an indication for choosing the right solvent.
- Since *dn/dc* is part of the equation of static light scattering, experimentally determining this parameter as precisely as possible is recommended.
- Sample fractionation is recommended to ensure that monodisperse samples are obtained and the *R_g_*, *R_h_*, *A_2_*, *M_w_*, and *dn*/*dc* values are determined with increased accuracy.
- When the light scattering detector is part of a chromatographic system, it is desired in the future to design new materials for chromatographic columns that can withstand for a long time the action of organic solvents usually used to dissolve cellulosic substances.
- If the intention is to compare the data obtained by several laboratories using laser light scattering or to confirm the data with those provided by other characterization methods, using the same experimental variables like solvent or the wavelength of the incident light would be ideal.
- Being biodegradable and biocompatible, cellulose-based materials are safely applied to living organisms. The practical applications of light scattering methods concern wastewater treatment, the pharmaceutical industry, the personal and healthcare industry, food-packing materials, and the restoration of heritage objects. To begin with, researchers were interested in light scattering studies regarding the solubility of cellulosic substances in various solvents and the phenomena of aggregation or self-assembly in solution. Other light scattering research has focused on the influence of different chemical and physical treatments on aging and implicitly on the depolymerization of cellulosic materials. Also, laser light scattering studies contributed to the elucidation of the interactions of cellulosic materials with various dispersants, plasticizers, surfactants, or inorganic particles in pharmaceutical and cosmetic formulas or drug delivery systems. Cellulose-based materials used as defoaming surfactants, adsorbents, and flocculants for pollutants, platforms for nanoparticle synthesis, and food packaging were also analyzed using laser light scattering.

## Figures and Tables

**Table 1:** Experimental results of cellulose solution studies in different compositions of NaOH and urea aqueous solvent systems at 25 °C [77].

Sample	*A_single_*/*A_agg_*	*M_w_*_,*app*_ (×10^4^ g/mol)	Ω*_single_*	*N_agg_*
Na_7_Ur_12_	0.32	19.6	0.63	5.5
Na_8_Ur_12_	0.43	16.3	0.67	4.6
Na_9_Ur_12_	0.84	10.6	0.86	4.1
Na_12_Ur_12_	-	139	-	-
Na_7_Ur_13_	0.34	18.9	0.65	5.4
Na_7_Ur_15_	0.33	18.8	0.65	5.4
Na_9_Ur_13_	1.40	8.1	0.96	3.6

**Table 2:** Particle size distributions and colloidal stability of cellulose nanocrystalline (CNC) particles in different electrolytes [79].

Type of Electrolytes		Ionic Strength	Particle Size (nm)	Zeta Potential (mV)	Interpretation of Data
Inorganic electrolytes	Na^+^/NaCl	2.5 or 5.0 mM	118–120	−38 to −32	Electrostatic screening effect of Na^+^
		10 mM–50 mM	151–980	−25.8 to −16.5	Particle aggregation
	Ca^2+^/CaCl_2_	≤1 mM	116–119	>30 mV	Little aggregation
		2.5–5.0 mM	325–752	−15.6 to −7.8	Particles tended to aggregate
Organic electrolytes	Anionic sodium dodecyl sulfate (SDS)	0.0–5.0 g/L	116–119	−55 to −75	Electrostatic repulsion between negatively charged SDS and CNCs
		15.0 g/L	105	−75	Low absorption of SDS on CNC surface
	Anionic sodium carboxymethyl cellulose (CMC)	0.0–2.5 g/L	100–600	−55 to −105	Larger aggregates
	Cationic poly(acrylamide) (CPAM)	0.5 g/L	-	-	Started aggregation

**Table 3:** Dimensions and zeta potential of CNCs extracted from HPMC, MCC, and CMC [87].

Sample	Length (*L*) (nm)		Average Width (*d*) (nm)	Average Aspect Ratio (*L*/*d*)	Zeta Potential (mV)
	SEM	DLS	SEM		
CNC_HPMC_	300–400	260	50–100	3–8	−8.6
CNC_MCC_	1000	820	100–300	3.3–10	−51.5
CNC_CMC_	300–600	218	50–100	3–6	−33.2

**Table 4:** Molecular weights and polydispersity indices for cellulose acetate phthalates determined in 8/2 (*v*/*v*) acetone–water containing different amounts of LiCl [92].

CAP Sample	LiCl Content (M)			
	0.005		0.0075	
	*M_w_*	*M_w_/M_n_*	*M_w_*	*M_w_/M_n_*
Wako	56,900	1.5	53,300	1.6
Eastman	54,400	1.6	54,700	1.7

## Data Availability

Not applicable.

## References

[1] Poletto M., Heitor L.O., Zattera A.J. (2014). Native cellulose: Structure, characterization and thermal properties. Materials.

[2] Jonoobi M., Oladi R., Davoudpour Y., Oksman K., Dufresne A., Hamzeh Y., Davoodi R. (2015). Different preparation methods and properties of nanostructured cellulose from various natural resources and residues: A review. Cellulose.

[3] Eichhorn S.J., Dufresne A., Aranguren M., Marcovich N.E., Capadona J.R., Rowan S.J., Weder C., Thielemans W., Roman M., Renneckar S. (2010). Review: Current international research into cellulose nanofibers and nanocomposites. J. Mater. Sci..

[4] Gatenholm P., Klemm D. (2010). Bacterial nanocellulose as a renewable material for biomedical applications. MRS Bull..

[5] Klemm D., Heublein B., Fink H.P., Bohn A. (2005). Cellulose: Fascinating biopolymer and sustainable raw material. Angew. Chem. Int. Ed..

[6] Ullah H., Santos H.A., Khan T. (2016). Applications of bacterial cellulose in food, cosmetics and drug delivery. Cellulose.

[7] Shokri J., Adibki K., van de Ven T., Godbout L. (2013). Application of cellulose and cellulose derivatives in pharmaceutical industries. Cellulose—Medical, Pharmaceutical and Electronic Applications.

[8] Sun Y., Wang J., Li D., Cheng F. (2024). The recent progress of the cellulose-based antibacterial hydrogel. Gels.

[9] Chen C., Xi Y., Weng Y. (2022). Recent advances in cellulose-based hydrogels for tissue engineering applications. Polymers.

[10] Nath P.C., Debnath S., Sharma M., Sridhar K., Nayak P.K., Inbaraj B.S. (2023). Recent advances in cellulose-based hydrogels: Food applications. Foods.

[11] Singh A.K., Itkor P., Lee Y.S. (2023). State-of-the-art insights and potential applications of cellulose-based hydrogels in food packaging: Advances towards sustainable trends. Gels.

[12] Nocca G., Arcovito A., Elkasabgy N.A., Basha M., Giacon N., Mazzinelli E., Abdel Maksoud M.S., Kamel R. (2023). Cellulosic textiles—An appealing trend for different pharmaceutical applications. Pharmaceutics.

[13] Omidian H., Akhzarmehr A., Chowdhury S.D. (2024). Advancements in cellulose-based superabsorbent hydrogels: Sustainable solutions across industries. Gels.

[14] Ma J., Li X., Bao Y. (2015). Advances in cellulose-based superabsorbent hydrogels. RSC Adv..

[15] Mikhailidi A., Volf I., Belosinschi D., Tofanica B.-M., Ungureanu E. (2023). Cellulose-based metallogels—Part 2: Physico-chemical properties and biological stability. Gels.

[16] Teng C.P., Tan M.Y., Toh J.P.W., Lim Q.F., Wang X., Ponsford D., Lin E.M.J., Thitsartarn W., Tee S.Y. (2023). Advances in cellulose-based composites for energy applications. Materials.

[17] Wang D.-C., Lei S.-N., Zhong S., Xiao X., Guo Q.-H. (2023). Cellulose-based conductive materials for energy and sensing applications. Polymers.

[18] Kulicke W.-M., Clasen C., Lohman C. (2005). Characterization of water-soluble cellulose derivatives in terms of the molar mass and particle size as well as their distribution. Macromol. Symp..

[19] Budtova T., Navard P. (2016). Cellulose in NaOH–water based solvents: A review. Cellulose.

[20] Dibrova A.K., Khanchich O. (2010). Cellulose solutions in dipolar aprotic solvents. Polym. Sci. Ser. A.

[21] Moon R.J., Martini A., Nairn J., Simonsenf J., Youngblood J. (2011). Cellulose nanomaterials review: Structure, properties and nanocomposites. Chem. Soc. Rev..

[22] Terinte N., Ibbett R., Schuster K.C. (2011). Overview on native cellulose and microcrystalline cellulose I structure studied by X-ray diffraction (WAXD): Comparison between measurements techniques. Lenzing. Berichte.

[23] Ummartyotin S., Manuspiya H. (2015). A critical review on cellulose: From fundamental to an approach on sensor technology. Renew. Sustain. Energy Rev..

[24] Lang P. Scattering Methods: Basic Principles and Application to Polymer and Colloidal Solutions, Part I, (Summer Term, 2004). https://www.yumpu.com/en/document/read/33367130/basic-principles-and-application-to-polymer-and-colloidal-solutions-.

[25] Braun B., Dorgan J.R., Chandler J.P. (2008). Cellulosic nanowhiskers. Theory and application of light scattering from polydisperse spheroids in the Rayleigh-Gans-Debye regime. Biomacromolecules.

[26] Gao W., Liu X.M., Gross R.A. (2009). Determination of molar mass and solution properties of cationic hydroxyethyl cellulose derivatives by multi-angle laser light scattering with simultaneous refractive index detection. Polym. Int..

[27] Podzimek S. (2011). Light Scattering, Size Exclusion Chromatography and Asymmetric Flow Field Flow Fractionation: Powerful Tools for Characterization of Polymers, Proteins and Nanoparticles.

[28] Schäertl W. (2007). Light Scattering from Polymer Solutions and Nanoparticle Dispersions.

[29] Potthast A., Rosenau T., Buchner R., Röder T., Ebner G., Bruglachner H., Sixta H., Kosma P. (2002). The cellulose solvent system N,N-dimethylacetamide/lithium chloride revisited: The effect of water on physicochemical properties and chemical stability. Cellulose.

[30] Lima M.M.D., Wong J.T., Paillet M., Borsali R., Pecora R. (2003). Translational and rotational dynamics of rodlike cellulose whiskers. Langmuir.

[31] van der Zande B.M.I., Dhont J.K.G., Bohmer M.R., Philipse A.P. (2000). Colloidal dispersions of gold rods characterized by dynamic light scattering and electrophoresis. Langmuir.

[32] de la Torre J.G., Carrasco B. (1998). Intrinsic viscosity and rotational diffusion of bead models for rigid macromolecules and bioparticles. Eur. Biophys. J..

[33] Broersma S. (1960). Rotational diffusion constant of a cylindrical particle. J. Chem. Phys..

[34] Broersma S. (1981). Viscous force and torque constants for a cylinder. J. Chem. Phys..

[35] de la Torre J.G., Bloomfield V.A. (1981). Hydrodynamic properties of complex, rigid, biological macromolecules: Theory and applications. Q. Rev. Biophys..

[36] Dreux M., Lafosse M., El Rassi Z. (1995). Evaporative light scattering detection of carbohydrates in HPLC. Carbohydrate Analysis: High Performance Liquid Chromatography and Capillary Electrophoresis.

[37] Douville V., Lodi A., Miller J., Nicolas A., Clarot I., Prilleux B., Megoulas N., Koupparis M. (2006). Evaporative light scattering detection (ELSD): A tool for improved quality control of drug substances. Pharmeur. Sci. Notes.

[38] Shakun M., Maier H., Heinze T., Kilz P., Radke W. (2013). Molar mass characterization of sodium carboxymethyl cellulose by SEC-MALLS. Carbohyd. Polym..

[39] Rashan J., Chen R. (2007). Developing a versatile gradient elution LC/ELSD method for analyzing cellulose derivatives in pharmaceutical formulations. J. Pharmaceut. Biomed. Anal..

[40] Wang S., Liu C., Chen Y., Zhang Z., Zhu A., Qian F. (2018). A high-sensitivity HPLC-ELSD method for HPMC-AS quantification and its application in elucidating the release mechanism of HPMC-AS based amorphous solid dispersions. Eur. J. Pharmaceut. Sci..

[41] (2002). ASTRA for Windows User’s Guide.

[42] Gamelas J.A.F., Pedrosa J., Lourenc A.F., Mutjé P., González I., Chinga-Carrasco G., Singh G., Ferreir P.J.T. (2015). On the morphology of cellulose nanofibrils obtained by TEMPO-mediated oxidation and mechanical treatment. Micron.

[43] Potthast A., Radosta S., Saake B., Lebioda S., Heinze T., Henniges U., Isogai A., Koschella A., Kosma P., Rosenau T. (2015). Comparison testing of methods for gel permeation chromatography of cellulose: Coming closer to a standard protocol. Cellulose.

[44] Jeong M.J., Bogolitsyna A., Jo B.M., Kang K.Y., Rosenau T., Potthast A. (2014). Deterioration of ancient Korean paper (Hanji), treated with beeswax: A mechanistic study. Carbohyd. Polym..

[45] Liu X.M., Gao W., Maziarz E.P., Salamone J.C., Duex J., Xia E. (2006). Detailed characterization of cationic hydroxyethylcellulose derivatives using aqueous size-exclusion chromatography with on-line triple detection. J. Chromatogr. A.

[46] Kacík F., Podzimek S., Vizarova K., Kacikova D., Cabalova I. (2016). Characterization of cellulose degradation during accelerated ageing by SEC-MALS, SEC-DAD, and A4F-MALS methods. Cellulose.

[47] Guan X., Cueto R., Russo P., Qi Y., Wu Q. (2012). Asymmetric flow field-flow fractionation with multiangle light scattering detection for characterization of cellulose nanocrystals. Biomacromolecules.

[48] Jiang Z., Lu A., Zhou J., Zhang L. (2012). Interaction between –OH groups of methylcellulose and solvent in NaOH/urea aqueous system at low temperature. Cellulose.

[49] Striegel A.M., Isenberg S.L., Cote G.L. (2009). An SEC/MALS study of alternan degradation during size-exclusion chromatographic analysis. Anal. Bioanal. Chem..

[50] Strlic M., Kolar J. (2003). Size exclusion chromatography of cellulose in LiCl/N,N-dimethylacetamide. J. Biochem. Bioph. Meth..

[51] Berggren R., Berthold F., Sjöholm E., Lindström M. (2003). Improved methods for evaluating the molar mass distributions of cellulose in Kraft pulp. J. Appl. Polym. Sci..

[52] Saito T., Yanagisawa M., Isogai A. (2005). TEMPO-mediated oxidation of native cellulose: SEC–MALLS analysis of water-soluble and -insoluble fractions in the oxidized products. Cellulose.

[53] Dupont A.-L., Harrison G. (2004). Conformation and d*n*/d*c* determination of cellulose in *N,N*-dimethylacetamide containing lithium chloride. Carbohyd. Polym..

[54] Yanagisawa M., Shibata I., Isogai A. (2004). SEC–MALLS analysis of cellulose using LiCl/1,3-dimethyl-2-imidazolidinone as an eluent. Cellulose.

[55] Yanagisawa M., Isogai A. (2005). SEC−MALS−QELS study on the molecular conformation of cellulose in LiCl/amide solutions. Biomacromolecules.

[56] Lojewski T., Zieba K., Lojewska J. (2010). Size exclusion chromatography and viscometry in paper degradation studies. New Mark-Houwink coefficients for cellulose in cupri-ethylenediamine. J. Chromatogr. A.

[57] Yamamoto M., Kuramae R., Yanagisawa M., Ishii D., Isogai A. (2011). Light-scattering analysis of native wood holocelluloses totally dissolved in LiCl–DMI solutions: High probability of branched structures in inherent cellulose. Biomacromolecules.

[58] Kes M., Christensen B.E. (2013). Degradation of cellulosic insulation in power transformers: A SEC–MALLS study of artificially aged transformer papers. Cellulose.

[59] Pawcenis D., Thomas J.L., Lojewski T., Milczarek J.M., Lojewska J. (2015). Towards determination of absolute molar mass of cellulose polymer by size exclusion chromatography with mulitple angle laser light scattering detection. J. Chromatogr. A.

[60] Ahmad W., Kuitunen S., Sixta H., Alopaeus V. (2015). Population balance based modeling of changes in cellulose molecular weight distribution during ageing. Cellulose.

[61] Hiraoki R., Fukuzumi H., Ono Y., Saito T., Isogai A. (2014). SEC-MALLS analysis of TEMPO-oxidized celluloses using methylation of carboxyl groups. Cellulose.

[62] Ono Y., Tanaka R., Funahashi R., Takeuchi M., Saito T., Isogai A. (2016). SEC–MALLS analysis of ethylenediamine-pretreated native celluloses in LiCl/N,N-dimethylacetamide: Softwood Kraft pulp and highly crystalline bacterial, tunicate, and algal celluloses. Cellulose.

[63] Ono Y., Ishida T., Soeta H., Saito T., Isogai A. (2016). Reliable d*n*/dc values of cellulose, chitin, and cellulose triacetate dissolved in LiCl/*N,N*-dimethylacetamide for molecular mass analysis. Biomacromolecules.

[64] Aono H., Tatsumi D., Matsumoto T. (2006). Scaling analysis of cotton cellulose/LiCl·DMAc solution using light scattering and rheological measurements. J. Polym. Sci. Pol. Phys..

[65] Aono H., Tatsumi D., Matsumoto T. (2006). Characterization of aggregate structure in mercerized cellulose/LiCl·DMAc solution using light scattering and rheological measurements. Biomacromolecules.

[66] Mandal A., Chakrabarty D. (2011). Isolation of nanocellulose from waste sugarcane bagasse (SCB) and its characterization. Carbohyd. Polym..

[67] Lue A., Zhang L. (2010). Effects of carbon nanotubes on rheological behavior in cellulose solution dissolved at low temperature. Polymer.

[68] Zhang J., Cao Y., Feng J., Wu P. (2012). Graphene-oxide-sheet-induced gelation of cellulose and promoted mechanical properties of composite aerogels. J. Phys. Chem. C.

[69] Ishii D., Kanazawa Y., Tatsumi D., Matsumoto T. (2007). Effect of solvent exchange on the pore structure and dissolution behavior of cellulose. J. Appl. Polym. Sci..

[70] Zhao G.H., Kapur N., Carlin B., Selinger E., Guthrie J.T. (2011). Characterization of the interactive properties of microcrystalline cellulose-carboxymethyl cellulose hydrogels. Int. J. Pharm..

[71] Troshenkova S.V., Sashina E.S., Novoselov N.P., Arndt K.-F. (2010). Light scattering in diluted solutions of cellulose and hydroxypropylcellulose in 1-ethyl-3-methylimidazolium acetate. Russ. J. Gen. Chem..

[72] Chen Y., Zhang Y., Ke F., Zhou J., Wang H., Liang D. (2011). Solubility of neutral and charged polymers in ionic liquids studied by laser light scattering. Polymer.

[73] Boluk Y., Danumah C. (2014). Analysis of cellulose nanocrystal rod lengths by dynamic light scattering and electron microscopy. J. Nanopart. Res..

[74] Khouri S., Shams M., Tam K.C. (2014). Determination and prediction of physical properties of cellulose nanocrystals from dynamic light scattering measurements. J. Nanopart. Res..

[75] Chen D., Ven T.G.M. (2016). Morphological changes of sterically stabilized nanocrystalline cellulose after periodate oxidation. Cellulose.

[76] Zoppe J.O., Johansson L.S., Seppala J. (2015). Manipulation of cellulose nanocrystal surface sulfate groups toward biomimetic nanostructures in aqueous media. Carbohyd. Polym..

[77] Qin X., Lu A., Cai J., Zhang L. (2013). Stability of inclusion complex formed by cellulose in NaOH/urea aqueous solution at low temperature. Carbohyd. Polym..

[78] do Nascimento J.H.O., Luz R.F., Galvao F.M.F., Melo J.D.D., Oliveira F.R., Ladchumananandasivam R., Zille A. (2015). Extraction and characterization of cellulosic nanowhisker obtained from discarded cotton fibers. Mater. Today-Proc..

[79] Zhong L., Fu S., Peng X., Zhan H., Sun R. (2012). Colloidal stability of negatively charged cellulose nanocrystalline in aqueous systems. Carbohyd. Polym..

[80] Zhang C., Kang H., Li P., Liu Z., Zhang Y., Liu R., Xiang J.-F., Huang Y. (2016). Dual effects of dimethylsulfoxide on cellulose solvating ability of 1-allyl-3-methylimidazolium chloride. Cellulose.

[81] Rinaldi R. (2011). Instantaneous dissolution of cellulose in organic electrolyte solutions. Chem. Commun..

[82] Lin L.Z., Yamaguchi H., Suzuki A. (2013). Dissolution of cellulose in the mixed solvent of [bmim]Cl-DMAc and its application. RSC Adv..

[83] Bardet R., Belgacem N., Bras J. (2015). Flexibility and color monitoring of cellulose nanocrystal iridescent solid films using anionic or neutral polymers. ACS Appl. Mater. Interfaces.

[84] Engel P., Hein L., Spiess A. (2012). Derivatization-free gel permeation chromatography elucidates enzymatic cellulose hydrolysis. Biotechnol. Biofuels.

[85] Rein D.M., Khalfin R., Szekely N., Cohen Y. (2014). True molecular solutions of natural cellulose in the binary ionic liquid-containing solvent mixtures. Carbohyd. Polym..

[86] Zhou J., Zhang L., Cai J. (2004). Behavior of cellulose in NaOH/urea aqueous solution characterized by light scattering and viscometry. J. Polym. Sci. Part B Polym. Phys..

[87] Alves L., Medronho B., Antunes F.E., Fernández-García M.P., Ventura J., Araújo J.P., Romano A., Lindman B. (2015). Unusual extraction and characterization of nanocrystalline cellulose from cellulose derivatives. J. Mol. Liq..

[88] Saake B., Zenker M., Stein A., Puls J. (2006). Studies on pre-hump and main fractions of cellulose-2,5-acetate in acetone. Cellulose.

[89] Ramos L.A., Morgado D.L., El Seoud O.A., da Silva V.C., Frollini E. (2011). Acetylation of cellulose in LiCl-N,N-dimethylacetamide: First report on the correlation between the reaction efficiency and the aggregation number of dissolved cellulose. Cellulose.

[90] Ghareeb H.O., Malz F., Kilz P., Radke W. (2012). Molar mass characterization of cellulose acetates over a wide range of high DS by size exclusion chromatography with multi-angle laser light scattering detection. Carbohyd. Polym..

[91] Tsunashima Y., Ikuno M., Onodera G., Horii F. (2006). Low-temperature dynamic light scattering. I. Structural reorganization and physical gel formation in cellulose triacetate/methyl acetate dilute solution at −99–45 °C. Biopolymers.

[92] Porsch B., Hillang I., Karlsson A., Sundelöf L.-O. (2002). Distribution analysis of cellulose acetate phthalate by ion-exclusion-moderated size exclusion chromatography. Carbohyd. Polym..

[93] (2003). Standard Practice for Dissolving Polymer Material. https://www.astm.org/d5226-98.html.

[94] Grigoras A.G., Olaru N. (2017). Solubility behavior of cellulose acetate butyrate in mixture of solvents. Rev. Chim. Buchar..

[95] Olaru N., Anghel N., Pascariu P., Ailiesei G. (2019). Synthesis and testing of cellulose acetate nicotinate as adsorbent for Rhodamine B dye. J. Appl. Polym. Sci..

[96] Grigoras A.G., Grigoras V.C. (2021). Investigation of cellulose derivatives in solution: Laser light scattering and gel permeation chromatography studies. Rom. J. Phys..

[97] Zhang K., Geissler A., Heinze T. (2015). Reversibly crystalline nanoparticles from cellulose alkyl esters via nanoprecipitation. Part. Part. Syst. Charact..

[98] Adden R., Melander C., Brinkmalm G., Knarr M., Engelhardt J., Mischnick P. (2009). The applicability of enzymes in cellulose ether analysis. Macromol. Symp..

[99] Goodwin D.J., Picout D.R., Ross-Murphy S.B., Holland S.J., Martini L.G., Lawrence M.J. (2011). Ultrasonic degradation for molecular weight reduction of pharmaceutical cellulose ethers. Carbohyd. Polym..

[100] Pfefferkorn P., Beister J., Hild A., Thielking H., Kulicke W.-M. (2003). Determination of the molar mass and the radius of gyration, together with their distributions for methylhydroxyethylcelluloses. Cellulose.

[101] Porsch B., Andersson M., Wittgren B., Wahlund K.-G. (2002). Molecular mass distribution analysis of ethyl(hydroxyethyl)cellulose by size-exclusion chromatography with dual light-scattering and refractometric detection. J. Chromatogr. A.

[102] Allabash S., Nicolai T., Benyahia L., Tassin J.-F., Chassenieux C. (2014). Evidence for the co-existence of interpenetrating permanent and transient networks of hydroxypropyl methyl cellulose. Biomacromolecules.

[103] Fettaka M., Issaadi R., Moulai-Mostefa N., Dez I., Cerf D.L., Picton L. (2011). Thermo sensitive behavior of cellulose derivatives in dilute aqueous solutions: From macroscopic to mesoscopic scale. J. Colloid. Interf. Sci..

[104] Marcelo G., Saiz E., Tarazona M.P. (2007). Determination of molecular parameters of hydroxyethyl and hydroxypropyl celluloses by chromatography with dual detection. J. Chromatogr. A.

[105] Dogsa I., Tomsic M., Orehek J., Benigar E., Jamnik A., Stopar D. (2014). Amorphous supramolecular structure of carboxymethyl cellulose in aqueous solution at different pH values as determined by rheology, small angle X-ray and light scattering. Carbohyd. Polym..

[106] Wittgren B., Stefansson M., Porsch B. (2005). Interactions between sodium dodecyl sulphate and non-ionic cellulose derivatives studied by size exclusion chromatography with online multi-angle light scattering and refractometric detection. J. Chromatogr. A.

[107] Lauten R.A., Nyström B. (2003). Time dependent association phenomena in dilute aqueous mixtures of a hydrophobically modified cellulose derivative and an anionic surfactant. Colloid. Surf. A.

[108] de Martins R.M., Silva C.A., Becker C.M., Samios D., Christoff M., Bica C.I.D. (2006). Interaction of (hydroxypropyl) cellulose with anionic surfactants in dilute regime. Colloid. Polym. Sci..

[109] de Martins R.M., Becker C.M., Samios D., Bica C.I.D. (2007). Interaction of (hydroxypropylmethyl)cellulose with anionic surfactants. Macromol. Symp..

[110] Beheshti N., Nguyen G.T.M., Kjøniksen A.-L., Knudsen K.D., Nyström B. (2006). Structure and dynamics of aqueous mixtures of an anionic cellulose derivative and anionic or cationic surfactants. Colloid. Surf. A.

[111] Chen R., Ilasi N., Sekulic S.S. (2011). Absolute molecular weight determination of hypromellose acetate succinate by size exclusion chromatography: Use of a multiangle laser light scattering detector and a mixed solvent. J. Pharmaceut. Biomed. Anal..

[112] Fischer K., Krasselt K., Schmidt I., Weightman D. (2005). Distribution of substituents along the cellulose chain on cellulose xanthate and carboxymethyl cellulose. Macromol. Symp..

[113] Li Q., Wu P., Zhou J., Zhang L. (2012). Structure and solution properties of cyanoethyl celluloses synthesized in LiOH/urea aqueous solution. Cellulose.

[114] Song Y., Zhang L., Gan W., Zhou J., Zhang L. (2011). Self-assembled micelles based on hydrophobically modified quaternized cellulose for drug delivery. Colloid. Surf. B.

[115] Li H., Wu B., Mu C., Lin W. (2011). Concomitant degradation in periodate oxidation of carboxymethyl cellulose. Carbohyd. Polym..

[116] Tan H., Wu B., Li C., Mu C., Li H., Lin W. (2015). Collagen cryogel cross-linked by naturally derived dialdehyde carboxymethyl cellulose. Carbohyd. Polym..

[117] Demeter J., Mormann W., Schmidt J., Burchard W. (2003). Solution behavior of trimethylsilyl cellulose of different degrees of substitution, studied by static and dynamic light scattering. Macromolecules.

[118] Mhlanga S.S. (2011). Investigating the Relative Adsorption of Polymeric Depressants on Pure Minerals. Master’s Thesis.

[119] Parolis L.A.S., van der Merwe R., Groenmeyer G.V., Harris P.J. (2008). The influence of metal cations on the behavior of carboxymethyl celluloses as talc depressants. Colloid. Surf. A.

[120] Smiechowicz E., Kulpinski P., Niekraszewicz B., Bacciarelli A. (2011). Cellulose fibers modified with silver nanoparticles. Cellulose.

[121] Sharma P.R., Kamble S., Sarkar D., Anand A., Varma A.J. (2016). Shape and size engineered cellulosic nanomaterials as broad spectrum anti-microbial compounds. Int. J. Biol. Macromol..

[122] Rowe R.C., Sheskey P.J., Owen S.C. (2006). Handbook of Pharmaceutical Excipients.

[123] Shukla R.K., Tiwari A. (2012). Carbohydrate polymers: Applications and recent advances in delivering drugs to the colon. Carbohyd. Polym..

[124] Ramesh Babu V., Krishna Rao K.S.V., Lee Y.I. (2010). Preparation and characterization of nifedipine-loaded cellulose acetate butyrate based microspheres and their controlled release behavior. Polym. Bull..

[125] Rokhade A.P., Agnihotri S.A., Patil S.A., Mallikarjuna N.N., Kulkarni P.V., Aminabhavi T.M. (2006). Semi-interpenetrating polymer network microspheres of gelatin and sodium carboxymethyl cellulose for controlled release of ketorolac tromethamine. Carbohyd. Polym..

[126] Grishkewich N., Mohammed N., Tang J., Tam K.C. (2017). Recent advances in the application of cellulose nanocrystals. Curr. Opin. Colloid. Interface Sci..

[127] Yu H., Yan C., Yao J. (2014). Fully biodegradable food packaging materials based on functionalized cellulose nanocrystals/poly(3-hydroxybutyrate-co-3-hydroxyvalerate) nanocomposites. RSC Adv..

[128] Cheng S., Zhang Y., Cha R., Yang J., Jiang X. (2016). Water-soluble nanocrystalline cellulose films with highly transparent and oxygen barrier properties. Nanoscale.

[129] Ioelovich M., Figovsky O. (2010). Structure and properties of nanoparticles used in paper compositions. Mech. Compos. Mater..

